# Dimers of pyrrolo-annelated indenofluorene-extended tetrathiafulvalenes – large multiredox systems†

**DOI:** 10.1039/d0ra02787a

**Published:** 2020-04-16

**Authors:** Line Broløs, Mogens Brøndsted Nielsen

**Affiliations:** Department of Chemistry, University of Copenhagen, Universitetsparken 5 DK-2100 Copenhagen Ø Denmark mbn@chem.ku.dk

## Abstract

Novel scaffolds of indenofluorene (IF)-extended tetrathiafulvalenes (TTF) were synthesized starting from a new pyrrolo-annelated IF-TTF monomer. Rigid *para*- and *meta*-phenylene linked dimers were obtained *via N*-arylation reactions of the monomer, and their optical and redox properties were elucidated by UV-Vis absorption spectroscopy and cyclic and differential pulse voltammetries.

## Abstract 2

A new pyrrolo-annelated indenofluorene-extended tetrathiafulvalene building block was developed and employed in *N*-arylation reactions for construction of redox-active dimeric scaffolds.

## Graphical abstract

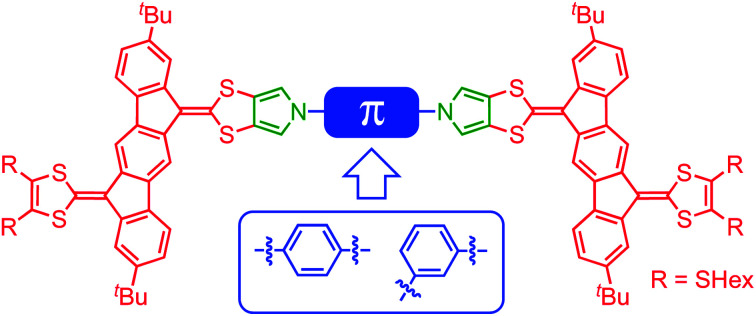

Tetrathiafulvalene (TTF) is a redox-active unit that reversibly undergoes two sequential one-electron oxidations, forming first a radical cation (TTF^+^) and subsequently a dication (TTF^2+^) containing two aromatic 1,3-dithiolium rings, and it is due to these redox properties that it is an attractive unit for materials and supramolecular chemistries.^1^

Extension of the conjugated system, leading to so-called extended TTFs, has successfully been used as a tool to finely tune the redox properties and geometries of the various redox states.^2^ For example, introduction of an indeno[1,2-*b*]fluorene (IF) core^3^ has provided indenofluorene-extended TTFs of the general structure IF-TTF shown in Fig. 1. X-Ray crystallographic and computational studies reveal that all three redox states (0, +1, +2), generated in sequential and reversible steps, take a fully planar structure, and spectroelectrochemical studies have shown that the individual redox states exhibit significantly redshifted absorptions relative to those of TTF, TTF^+^, and TTF^2+^, respectively.^2^

**Fig. 1:**
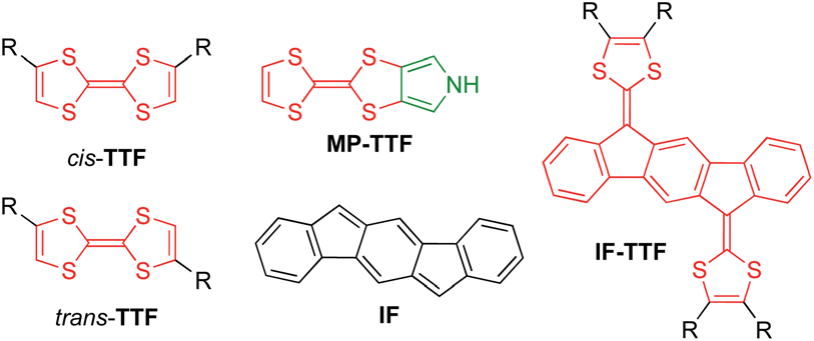
Structures of *cis*/*trans*-isomeric TTFs, mono-pyrrolo-TTF (MP-TTF), indenofluorene (IF) and an indenofluorene-extended TTF (IF-TTF).

Recently, we developed synthetic protocols for linking together two IF-TTF units *via* anchoring at a peripheral position of each dithiafulvene unit.^4^ Such dimers unfortunately exist as unseparable mixtures of *cis* and *trans* isomers (*cf.*, the disubstituted TTFs shown in Fig. 1), and to avoid this problem of isomerism we decided to develop a synthetic protocol for fusing a pyrrole unit to one of the dithiole rings of IF-TTF as in target molecule 1 shown in Fig. 2. Indeed, the related mono-pyrrolo-annelated TTF (MP-TTF, Fig. 1) and bis-pyrrolo-annelated TTF have proven important as versatile π-donor building blocks in macromolecular and supramolecular chemistry.^5^

**Fig. 2:**
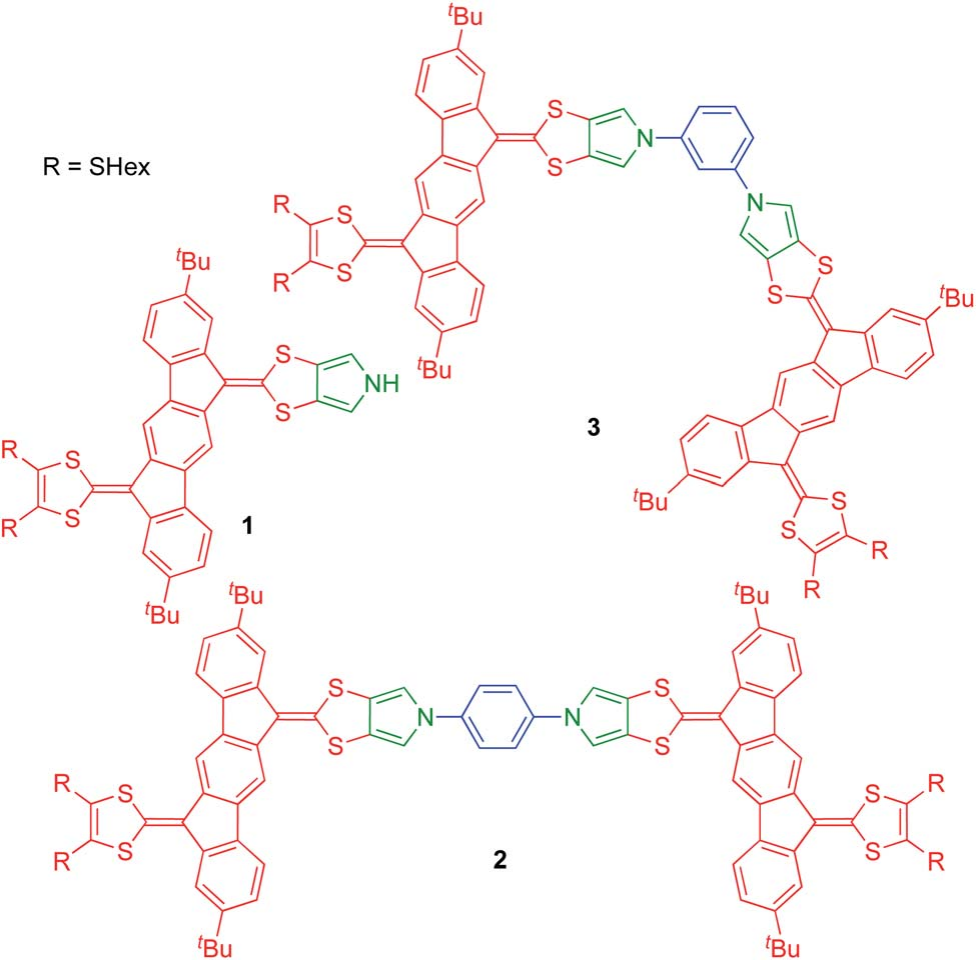
New pyrrolo-annelated IF-TTF mono- and dimers.

Dimerization of two units 1*via* its nitrogen atom and a suitable linker would prevent formation of isomers. As linkers we decided to explore rigid phenylene units as in target molecules 2 (*para*-phenylene bridge) and 3 (*meta*-phenylene bridge) shown in Fig. 2. Previously prepared *cis*/*trans* isomeric IF-TTF dimers had flexible linkers and showed intramolecular associations upon oxidation,^4^ which would be prevented by these rigid linkers. Moreover, intermolecular interactions are to a large extent prevented by the peripheral *tert*-butyl substituents that were chosen as substituent groups to enhance solubility of the dimers.

Synthesis of 1 proceeds according to Scheme 1, employing the known diketone 4,^4^ the *N*-tosyl-protected pyrrolo-annelated 1,3-dithiole-2-thione I^5*a*^ and the phosphonate ester II^3*b*^ as precursors. A phosphite-mediated coupling between 4 and I was carried out to give mono-olefinated product 5 in a yield of 61%. This compound was next subjected to a Horner–Wadsworth–Emmons olefination with compound II, deprotonated by sodium hexamethyldisilazide (NaHMDS), providing the tosyl-protected mono-pyrrolo IF-TTF6 in good yield (76%). This compound was subsequently deprotected using NaOMe to give in almost quantitative yield the monopyrrolo IF-TTF1 with a pyrrole N–H unit available for further reactions.

**Scheme 1:**
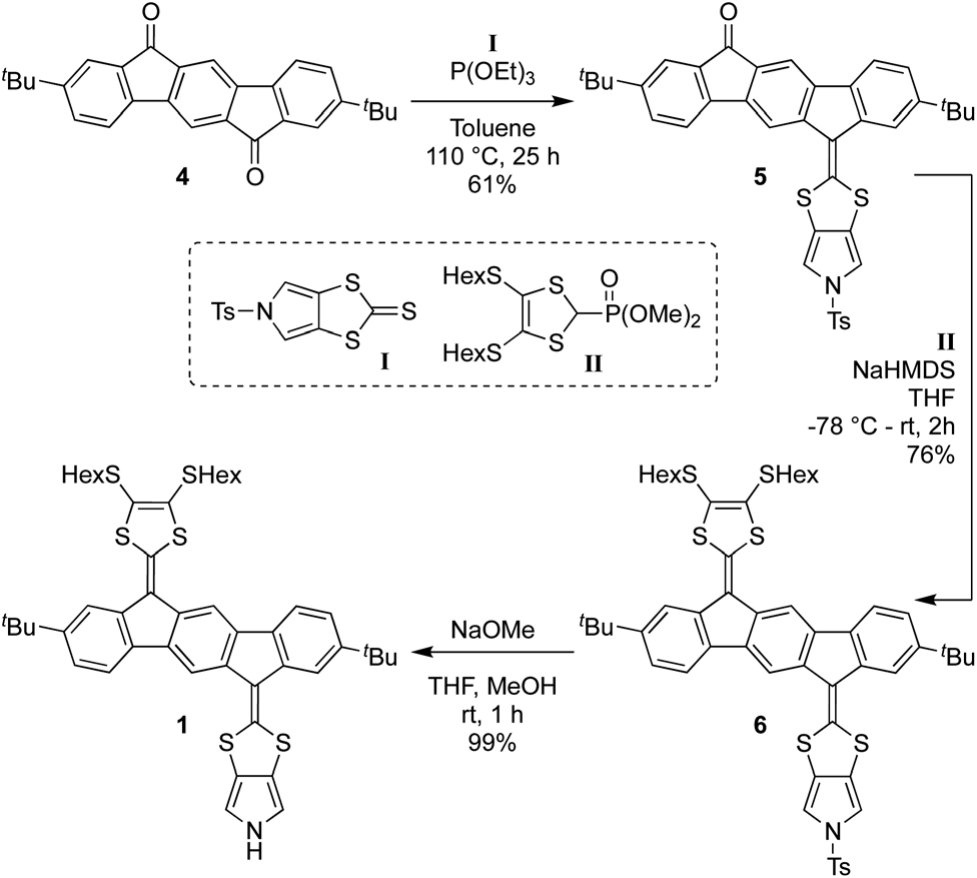
Synthesis of monomer 1 by stepwise olefination reactions. NaHMDS = sodium hexamethyldisilazide.

With monomer 1 in hand, *N*-arylation reactions with 1,4- and 1,3-diiodobenzene, catalyzed by an excess of CuI and (±)-1,2-*trans*-diaminocyclohexane, were conducted in THF at reflux (Scheme 2). This procedure, previously applied for *N*-arylation of pyrrolo-annelated TTFs,^6^ yielded dimers 2 and 3, respectively. The procedure worked best for 1,3-diiodobenzene. For the arylation reaction with 1,4-diiodobenzene it was observed that the first arylation progressed rather willingly, as the mono-arylated product 7 could be isolated in 91% yield after only 3 hours. Only when compound 7 was subjected to significantly longer reaction time, however, formation of dimer 2 was observed, and the compound was isolated in 9% yield after 18 hours of reaction time. This result indicates that the substitution of an iodide on the benzene ring with one IF-TTF unit, in the *para* position, decreases the reactivity of the second iodide significantly, possibly due to the strongly electron-donating character of the pyrrolo-TTF. Albeit inconvenient in the current work, it could be a potential advantage for stepwise construction of unsymmetrical scaffolds. For the corresponding reaction with 1,3-diiodobenzene smooth formation of the dimer 3 was observed, and this product was isolated in 23% after 16 hours, while no mono-arylated intermediate could be isolated.

**Scheme 2:**
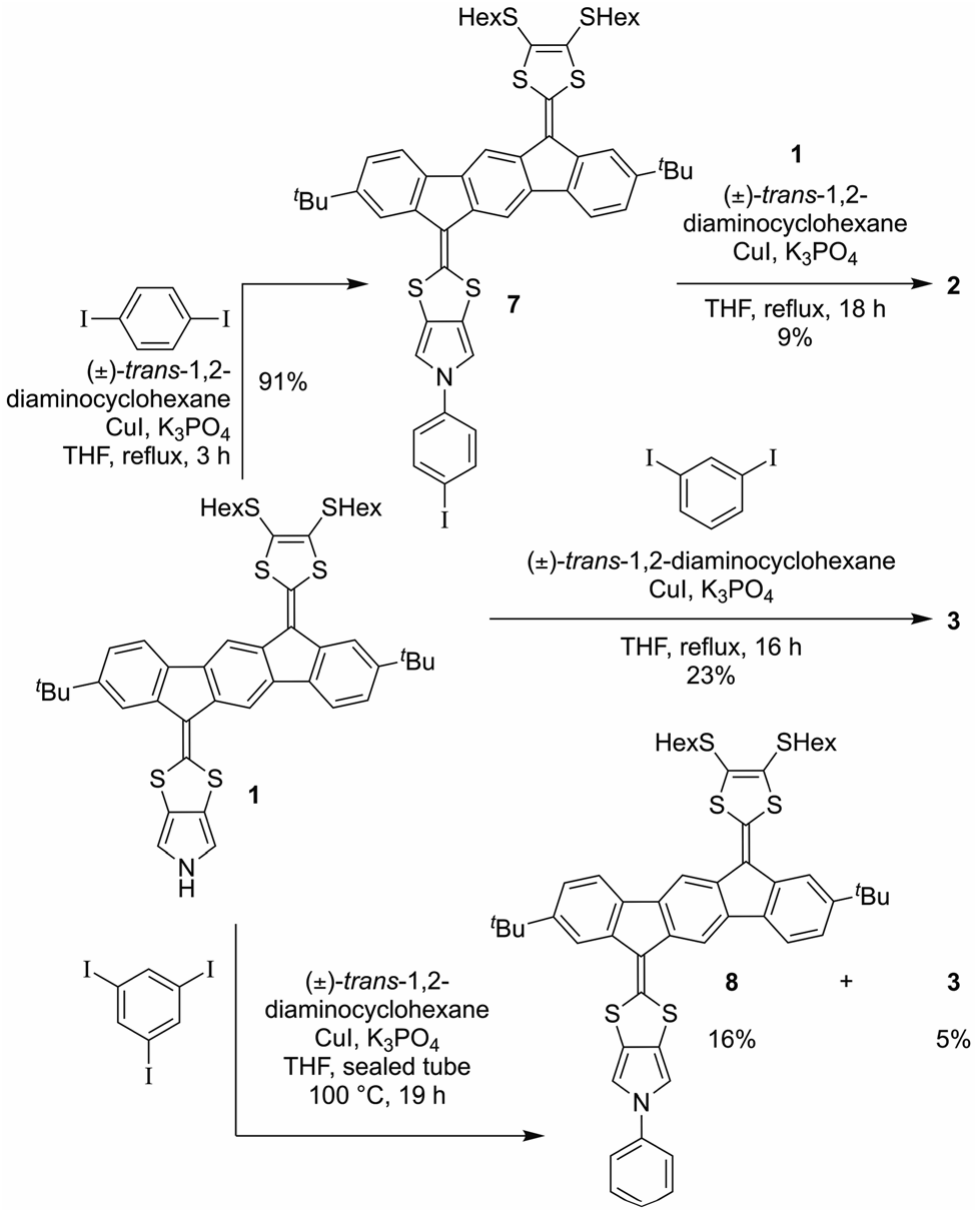
Synthesis of dimers 2 and 3, and of mono-arylated species 7 and 8.

Synthesis of a trimer consisting of three IF-TTF units around one central benzene ring was also attempted by coupling of monomer 1 and 1,3,5-triiodobenzene. However, when employing the conditions proven successful for synthesis of the dimers, no reaction was observed; instead, 86% of the starting material was re-isolated. Nevertheless, when conducting the reaction in a sealed vial and heating to 100 °C, full conversion of monomer 1 was observed. However, the isolated products were mono-arylated monomer 8 and previously isolated dimer 3, and not the desired trimer. This result signals again that the reactivity of the iodides in the arylation reaction decreases upon introduction of IF-TTF units. Upon elevated pressure, as applied in the attempt to achieve the desired trimer, a competing reaction by which the iodides are substituted for hydrogen atoms is observed to exceed the arylation reaction. The mono-arylated compound 8 was used as a reference compound in subsequent studies of the synthesized dimers.

The photophysical properties of monomers 1 and 8 as well as dimers 2 and 3 were investigated by UV-Vis absorption spectroscopy in CH_2_Cl_2_ at 25 °C (Fig. 3). Absorption maxima and extinction coefficients are listed in Table 1. The longest-wavelength absorption maximum of 1 (468 nm) is close to that of the related IF-TTF with four peripheral SEt substituents (473 nm; R = SEt in Scheme 1).^3*a*^ Expansion of the π-system with a benzene ring (compound 8) had little effect on the longest-wavelength absorption maximum (471 nm). Linking the monomeric units by phenylene linkers, dimers 2 and 3, did not change the longest-wavelength absorption maxima significantly either, but the intensity of the absorption was expectedly doubled (or slightly more than doubled).

**Fig. 3:**
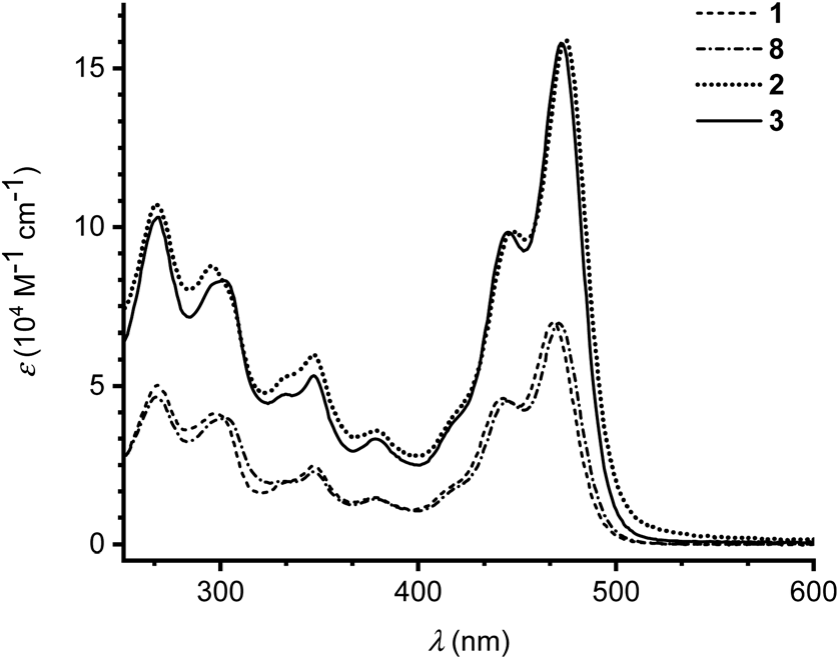
UV-Vis absorption spectra of monomer 1 (dashed line), monomer 8 (dot dash dot) and dimers 2 (dotted line) and 3 (full line) in CH_2_Cl_2_ at 25 °C.

**Table 1:** Absorption maxima (*λ*_max_) and extinction coefficients (*ε*) in CH_2_Cl_2_ at 25 °C, and oxidation potentials (from DPV) *E*_ox_*vs.* Fc/Fc^+^ in 1 : 1 CH_2_Cl_2_/C_6_H_5_Cl, for compounds 1, 2, 3 and 8

Compound	*λ*_max_/nm (*ε*/10^4^ M^−1^ cm^−1^)	*E*_ox_ (V *vs.* Fc/Fc^+^)
1	468 (6.99), 443 (4.61), 378 (1.47), 348 (2.46), 298 (4.13), 267 (5.00)	+0.18 (1e), +0.36 (1e)
2	475 (15.9), 448 (9.88), 379 (3.59), 347 (5.98), 295 (8.78), 267 (10.7)	+0.11 (1e), +0.44 (2e)
3	472 (15.8), 445 (9.84), 379 (3.32), 347 (5.32), 302 (8.31), 269 (10.3)	+0.14 (1e), +0.20 (1e), +0.41 (2e)
8	471 (6.97), 444 (4.52), 381 (1.41), 347 (2.29), 302 (3.98), 268 (4.65)	+0.22 (1e), +0.33 (1e)

Electrochemical studies of the synthesized compounds were conducted in 1 : 1 mixture of CH_2_Cl_2_/C_6_H_5_Cl containing 0.1 M NBu_4_PF_6_ as supporting electrolyte, and the cyclic voltammograms (CVs) and differential pulse voltammograms (DPVs) are shown in Fig. 4. Chlorobenzene was needed as co-solvent due to limited solubility of the dimers in neat CH_2_Cl_2_. Oxidation potentials are listed in Table 1 (taken from the DPVs), referenced against the ferrocene/ferrocenium (Fc/Fc^+^) redox couple (recorded in a separate experiment). For monomer 1 two reversible, one-electron oxidations were observed, at +0.18 and +0.36 V *vs.* Fc/Fc^+^, forming the radical cation and the dication, respectively. Similarly, monomer 8 was found to undergo two reversible one-electron oxidations at +0.22 and +0.33 V *vs.* Fc/Fc^+^. For dimer 3 two reversible, one-electron oxidations were observed, at +0.14 and +0.20 V *vs.* Fc/Fc^+^, forming the radical cation and the dication, respectively, followed by a reversible two-electron oxidation, at 0.41 V *vs.* Fc/Fc^+^, forming the tetracation. The electrochemistry of dimer 2 is, however, more complicated. It exhibits a reversible one-electron oxidation at +0.11 V *vs.* Fc/Fc^+^, hence at lower potential than for dimer 3 in accordance to the large linearly conjugated system provided by a *para*-phenylene bridge. The second oxidation seemed, however, to occur over a very broad potential range. As known from literature,^7^ the isolated 1,4-di(*N*-pyrrolyl)benzene unit itself undergoes an irreversible oxidation, and in the case of dimer 2 it seems that the redox properties are affected significantly by this structural unit of the molecule; dimer 2 thereby acts less like a ‘classical’ extended TTF. The CV may as well be complicated by intermolecular interactions despite the bulky *tert*-butyl groups present on the IF cores. A reversible two-electron oxidation, possibly due to formation of the tetracation or higher oxidation states, is observed at +0.44 V *vs.* Fc/Fc^+^, indicating that while the second oxidation wave is significantly broadened the reversibility is intact.

**Fig. 4:**
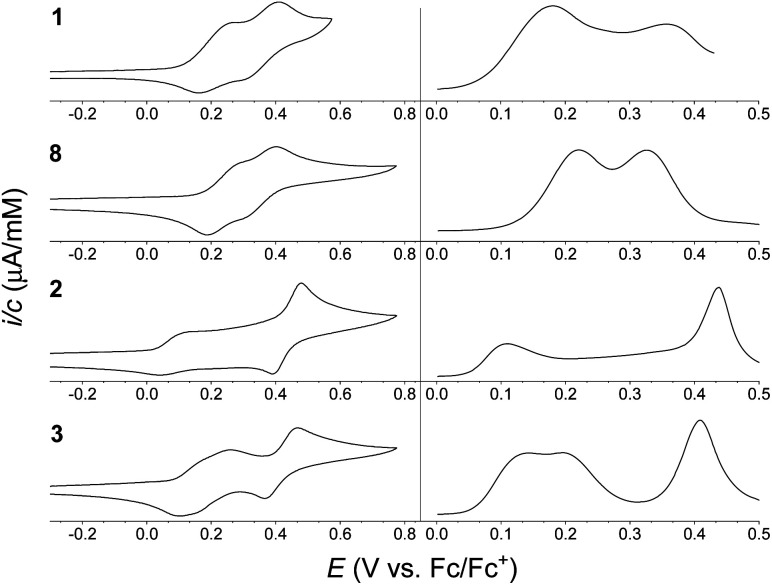
Cyclic voltammograms (CVs) (left) and differential pulse voltammograms (DPVs) (right) of (from the top) 1 (0.38 mM), 8 (0.38 mM), 2 (0.37 mM) and 3 (0.43 mM); potentials *vs.* Fc/Fc^+^ (solvent: 1 : 1 CH_2_Cl_2_/C_6_H_5_Cl; supporting electrolyte: 0.1 M NBu_4_PF_6_; scan rate: 0.1 V s^−1^). Oxidation potentials listed in Table 1 are based on DPVs.

In conclusion, we have developed a convenient synthetic procedure to obtain the first pyrrolo-annelated IF-extended TTF that was successfully dimerized by *N*-arylation reactions. The resulting rigid dimers present new interesting multiredox systems to be explored further in future work. The possibility to perform functionalization at the α-carbon atoms of the pyrrole unit of these new scaffolds will also be interesting to pursue, taking advantage of the elaborate chemistry that has been developed for the parent mono- and bis-pyrrolo-annelated TTFs.^5^

## Conflicts of interest

There are no conflicts to declare.

## Supplementary Material

RA-010-D0RA02787A-s001
