# Correction: Dimers of pyrrolo-annelated indenofluorene-extended tetrathiafulvalenes – large multiredox systems

**DOI:** 10.1039/d1ra90172a

**Published:** 2022-01-14

**Authors:** Line Broløs, Mogens Brøndsted Nielsen

**Affiliations:** Department of Chemistry, University of Copenhagen Universitetsparken 5 DK-2100 Copenhagen Ø Denmark mbn@chem.ku.dk

## Abstract

Correction for ‘Dimers of pyrrolo-annelated indenofluorene-extended tetrathiafulvalenes – large multiredox systems’ by Line Broløs *et al.*, *RSC Adv.*, 2020, **10**, 15030–15033, DOI: 10.1039/D0RA02787A.

The authors regret that the yield of dimer 3 was incorrectly reported as 23% in the original article. The correct yield is 58%. To reflect this, the following changes are needed.

The final sentence on the 2^nd^ page of the article should read “For the corresponding reaction with 1,3-diiodo-benzene smooth formation of the dimer 3 was observed, and this product was isolated in 58% after 16 hours, while no mono-arylated intermediate could be isolated.”

The yield should be updated on Scheme 2:

**Scheme 2:**
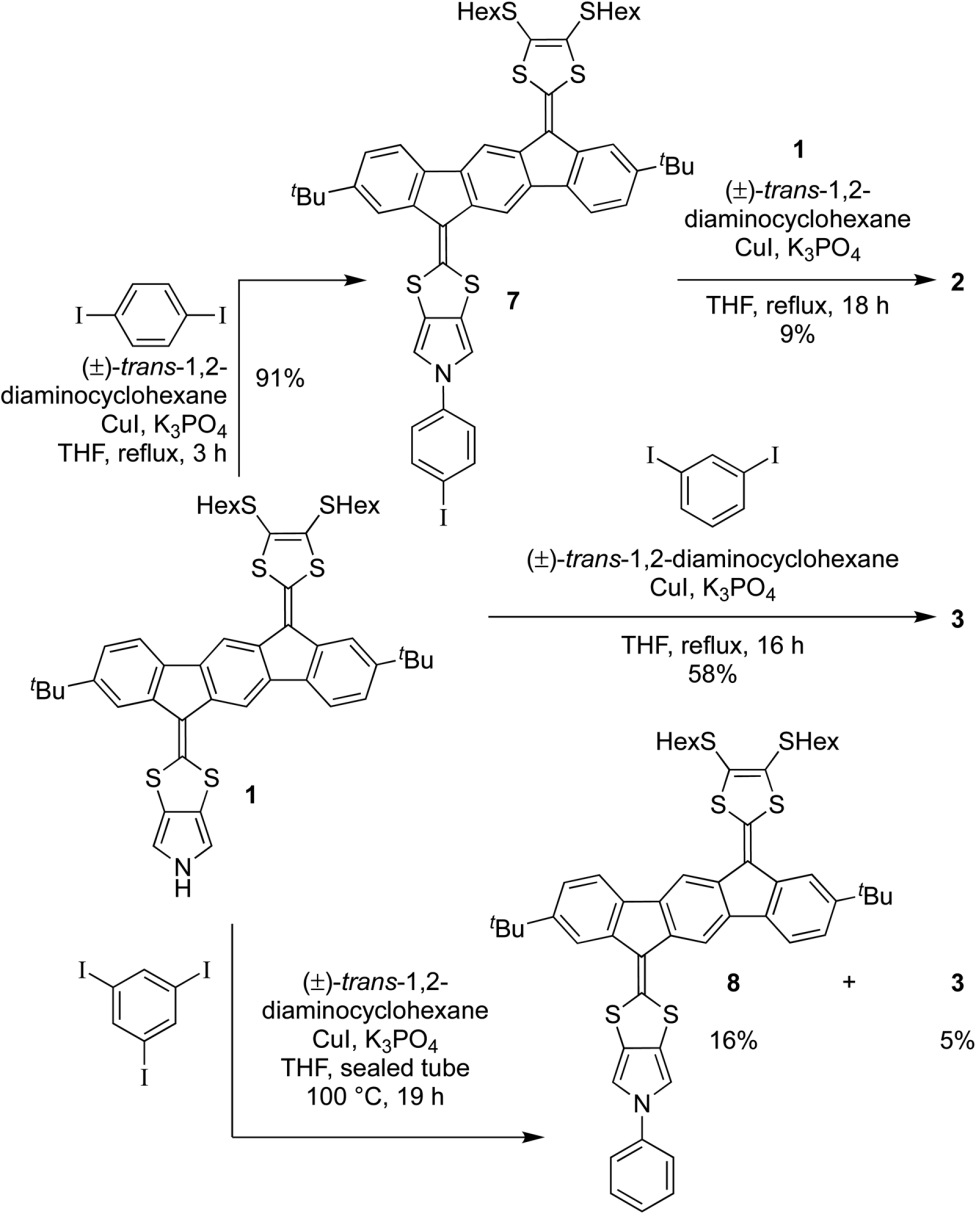


The ESI document has also been updated accordingly.

The Royal Society of Chemistry apologises for these errors and any consequent inconvenience to authors and readers.

## Supplementary Material

