# Supplementary material for: Dimers of pyrrolo-annelated indenofluorene-extended tetrathiafulvalenes – large multiredox systems
Source: RSC Adv. 2020 Apr 16;10(26):15030–3. doi: 10.1039/d0ra02787a (PMC9052312; doi:10.1039/d0ra02787a)
Supplement: RA-010-D0RA02787A-s001 [file RA-010-D0RA02787A-s001.pdf]

Electronic Supplementary Material (ESI) for RSC Advances.
This journal is © The Royal Society of Chemistry 2021

Dimers of pyrrolo-annelated indenofluorene-extended tetrathiafulvalenes – large multiredox systems

Line Broløs and Mogens Brøndsted Nielsen*

Department of Chemistry, University of Copenhagen, Universitetsparken 5, DK-2100 Copenhagen Ø, Denmark. E-mail: mbn@chem.ku.dk

Electronic Supplementary Information

Contents

General methods S2

Synthetic procedures for all new compounds S3

NMR spectra of all synthesized compounds S9

Full UV-Vis spectra S16

Electrochemical studies S17

General methods

Anhydrous MeOH was obtained by drying over 3Å sieves. All remaining anhydrous solvents were obtained from a solvent drying tower (IT model PS-MD-05). HPLC solvents were used. Elevated reaction temperatures were obtained by use of an aluminum heating mantle. Purification by chromatography was performed using silica gel (flash: 40-63 µm, dry: 15-40 µm). TLC was performed using aluminum sheets covered with silica gel coated with fluorescent indicator. NMR spectra were recorded on at 500 MHz and 126 MHz for ^{1}H- and ^{13}CNMR, respectively (using an instrument with a cryoprobe). Deuterated chloroform ($CDCl_3$, δ_H = 7.26 ppm, δ_C = 77.16 ppm) was used as solvent (and reference for calibration). The $CDCl_3$ was filtered through activated Al_2O_3 prior to use. Chemical shift values are referenced to the ppm scale and coupling constants are expressed in Hertz (Hz). In ^{13}C-APT (Attached Proton Test) NMR spectra, negative signals correspond to CH and CH_3, positive signals correspond to C and CH_2. HRMS was performed using MALDI with dithranol as matrix, using an FT-ICR instrument equipped with a 7-T Magnet. Melting points are not corrected.

UV/Vis absorption spectra were recorded by scanning between 800 and 200 nm. All spectra were recorded in CH_2Cl_2 (HPLC grade) at 25 °C in a 1-cm quartz cuvette. The concentrations of the compounds were: 0.004 mM (**1**), 0.009 mM (**8**), 0.002 mM (**2**) and 0.003 mM (**3**).

Cyclic voltammograms (CV) and differential pulse voltammograms (DPV) were obtained using a scan rate of 0.1 V/s. A silver wire was used as the reference electrode, a Pt wire was used as the counter electrode, and a glassy-carbon disk electrode (3 mm) was used as the working electrode. The reference electrode was separated from the solution containing the substrate by a ceramic frit. Measured potentials were referenced to the ferrocene/ferrocenium (Fc/Fc^{+}) redox couple measured before and after the experiment on a 0.1 mM solution of Fc. All solutions were purged with solvent saturated argon prior to measurement. The concentrations of the electroactive compounds were: 0.38 mM (**1**), 0.38 mM (**8**), 0.37 mM (**2**) and 0.43 mM (**3**). The voltammograms of the compounds were recorded in 1:1 mixture of CH_2Cl_2/C_6H_5Cl (HPLC grade) containing 0.1 M Bu_4NPF_6 supporting electrolyte. The iR-compensation was applied in order to compensate for the solvent resistance.

Synthetic procedures for all new compounds

Compound 1

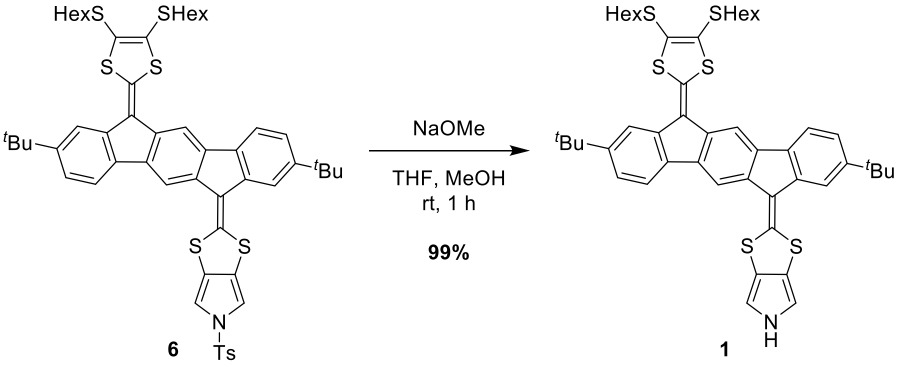

A solution of NaOMe - Na(s) (35 mg, 1.52 mmol) in anhydrous MeOH (1 mL) - was stirred at rt for 0.5 h before it was added in one portion to a solution of **6** (54 mg, 0.054 mmol) in anhydrous THF (10 mL) and anhydrous MeOH (10 mL). The mixture was stirred at rt for a further 1 h before H_2O (10 mL) was added, and 1 M HCl was added dropwise until pH of the solution was acidic. The solution was now diluted with CH_2Cl_2 (50 mL), washed with H_2O (3 x 20 mL), dried over $MgSO_4$, filtered and concentrated in vacuum. Purification by flash column chromatography (SiO_2, 10-30% EtOAc/heptane) yielded **1** (45 mg, 99%) as a yellow solid. ^{1}H NMR (500 MHz, $CDCl_3$) δ 8.47 (s, 1H), 8.43 (s, 1H), 8.11 (s, 1H), 8.10 (d, J = 1.6 Hz, 1H), 7.88 (dd, J = 9.8, 8.0 Hz, 2H), 7.78 (d, J = 1.6 Hz, 1H), 7.41 (ddd, J = 8.0, 6.8, 1.6 Hz, 2H), 6.91 – 6.89 (m, 2H), 3.03 – 2.96 (m, 4H), 1.79 – 1.73 (m, 4H), 1.49 (s, 9H), 1.46 (s, 9H), 1.36 – 1.31 (m, 12H), 0.92 – 0.87 (m, 6H) ppm. ^{13}C NMR (126 MHz, $CDCl_3$) δ 150.07, 149.98, 146.51, 138.24, 137.80, 137.42, 137.30, 136.45, 136.26, 135.98, 135.31, 135.25, 128.90, 128.29, 123.14, 123.09, 123.00, 121.95, 120.71, 120.26, 120.18, 120.11, 119.09, 118.89, 114.81, 114.11, 109.70, 109.67, 36.85, 36.79, 35.32, 35.24, 31.96, 31.91, 31.57, 31.51, 30.12, 30.01, 28.48, 28.47, 22.72, 22.72, 14.20, 14.18 ppm. HRMS (MALDI$^+$ FT-ICR, dithranol): m/z = 837.26398 $[M]^{\bullet+}$, calcd. for ($C_{13}H_{14}NO_5S_4^+$) m/z = 837.26533.

Compound 2

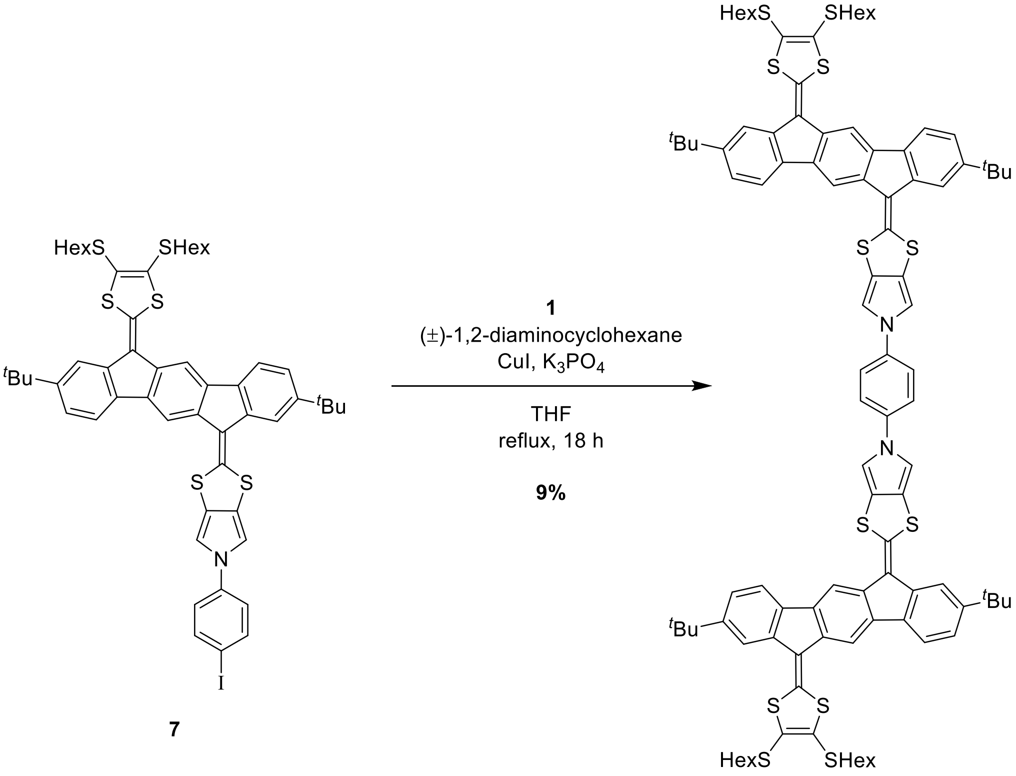

7

2

To a N_2 degassed solution of **7** (40 mg, 0.38 mmol) and **1** (30 mg, 0.36 mmol) in anhydrous THF (20 mL) were added CuI (29 mg, 0.15 mmol) and K_3PO_4 (45 mg, 210 mmol). The solution was further degassed with N_2 for 15 min. before (±)-*trans*-1,2-diaminocyclohexane (0.05 mL, 410 mmol) was added, and the mixture, undergoing a color change from yellow to red, was stirred at reflux for 18 h before it was cooled to rt, diluted with CH_2Cl_2 (50 mL), washed with H_2O (4 x 100 mL), dried over $MgSO_4$, filtered and concentrated in vacuum. Purification by flash column chromatography (SiO_2, 50% CH_2Cl_2/heptane) followed by size exclusion chromatography (SX3, CH_2Cl_2) and trituration of the precipitated solids with MeOH (3 x 4 mL) yielded **2** (5.9 mg, 9%) as a yellow solid. ^{1}H NMR (500 MHz, $CDCl_3$) δ 8.27 (s, 2H), 7.99 (d, J = 1.6 Hz, 2H), 7.98 (s, 2H), 7.79 (dd, J = 15.9, 8.0 Hz, 4H), 7.69 (d, J = 1.6 Hz, 2H), 7.42 – 7.35 (m, 8H), 7.03 (d, J = 7.0 Hz, 4H), 2.94 (t, J = 7.3 Hz, 4H), 2.89 (t, J = 7.3 Hz, 4H), 1.80 – 1.72 (m, 8H), 1.48 (s, 18H), 1.45 (s, 18H), 1.39 – 1.33 (m, 24H), 0.96 – 0.91 (m, 12H) ppm. ^{13}C NMR (126 MHz, $CDCl_3$) δ 149.53, 149.49, 144.57, 138.17, 137.98, 137.85, 137.51, 137.26, 136.41, 136.31, 135.76, 135.76, 135.51, 129.21, 128.39, 123.99, 123.40, 123.22, 123.18, 123.09, 122.03, 121.19, 120.83, 120.28, 119.10, 119.03, 114.98, 114.32, 110.52, 110.49, 37.08, 36.98, 35.05, 34.98, 32.01, 31.96, 31.91, 31.85, 30.34, 30.27, 28.86, 28.83, 23.24, 23.21, 14.58, 14.55 ppm. HRMS (MALDI$^+$ FT-ICR, dithranol): *m/z* = 1750.54511 [M+H]$^+$, calcd. For ($C_{102}H_{105}N_2O_4S_{12}^+$) *m/z* = 1750.55803.

Compound 3

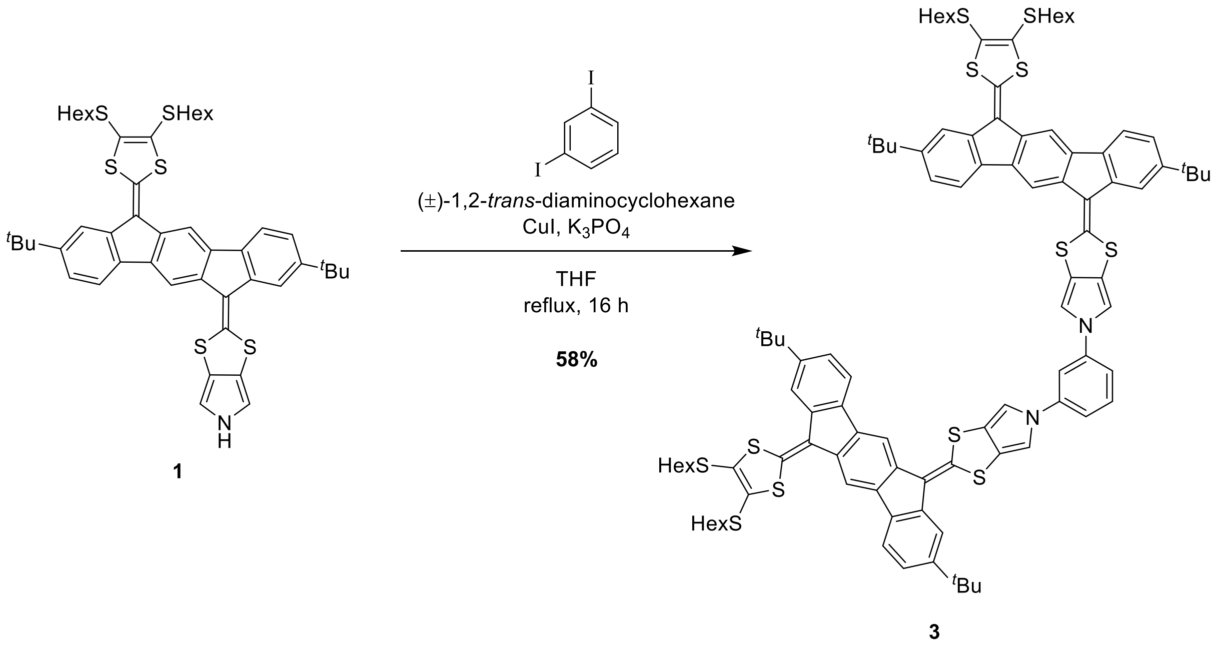

To a N_2 degassed solution of **1** (18 mg, 0.021 mmol) and 1,3-diiodobenzene (3.0 mg, 0.0091 mmol) in anhydrous THF (10 mL) were added CuI (35 mg, 0.18 mmol) and K_3PO_4 (20 mg, 0.12 mmol), and the mixture was further degassed with N_2 for 30 min. before (±)-*trans*-1,2-diaminocyclohexane (0.02 mL, 0.17 mmol) was added, and the mixture, undergoing a color change from yellow to red, was stirred at reflux for 16 h before it was cooled to rt, diluted with CH_2Cl_2 (50 mL), washed with H_2O (4 x 100 mL), dried over $MgSO_4$, filtered and concentrated in vacuum. Purification by flash column chromatography (SiO_2, 2-10% CH_2Cl_2/CS_2) yielded **3** (9.2 mg, 58%) as a yellow solid. ^{1}H NMR (500 MHz, $CDCl_3$) δ 8.31 (s, 2H H), 8.05 (d, J = 1.6 Hz, 2H), 8.00 (s, 2H), 7.84 (d, J = 7.9 Hz, 2H), 7.80 (d, J = 7.9 Hz, 2H), 7.72 (d, J = 1.6 Hz, 2H), 7.43 – 7.40 (m, 4H), 7.23 – 7.16 (m, 4H), 7.07 – 7.05 (d, J = 2.3 Hz, 4H), 3.00 (t, J = 7.4 Hz, 4H), 2.97 (t, J = 7.4 Hz, 4H), 1.79 – 1.73 (m, 8H), 1.54 (s, 18H), 1.50 (s, 18H), 1.41 – 1.32 (m, 24H), 1.03– 0.84 (m, 12H) ppm. ^{13}C NMR (126 MHz, $CDCl_3$) δ 149.63, 149.57, 144.48, 141.12, 138.06, 137.71, 137.38, 137.15, 136.30, 136.19, 135.65, 135.38, 135.38, 131.11, 128.86, 128.20, 123.85, 123.23, 123.19, 123.13, 123.06, 121.85, 120.70, 120.11, 118.96, 118.87, 117.07, 114.79, 114.11, 111.06, 110.33, 110.32, 36.85, 36.77, 35.03, 34.95, 31.87, 31.81, 31.67, 31.61, 30.14, 30.05, 28.61, 28.59, 22.93, 22.91, 14.30, 14.27. ppm. HRMS (MALDI$^+$ FT-ICR, dithranol): m/z = 1749.54606 $[M]^{\bullet+}$, calcd. For ($C_{102}H_{112}N_2S_{12}^{\bullet+}$) m/z = 1749.55021.

Compound 5

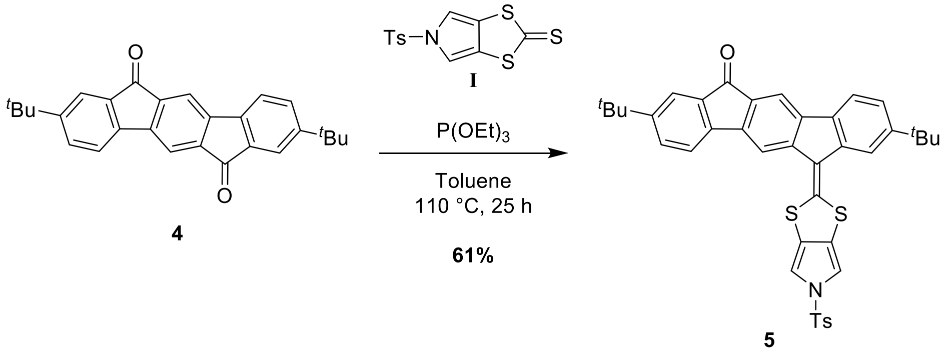

To a solution of **4** (222 mg, 0.563 mmol) in anhydrous toluene (10 mL) was added $P(OEt)_3$ (20 mL), and the mixture was heated to 110 °C after which **I** (350 mg, 1.07 mmol) was added, and the mixture was stirred for 25 h before it was concentrated in vacuum. Purification by flash column chromatography (SiO_2, 1) 50-70% CH_2Cl_2/heptane, 2) 20% EtOAc/heptane) yielded **5** (230 mg, 61%) as an orange solid. ^{1}H NMR (500 MHz, $CDCl_3$) δ 8.02 (d, J = 0.8 Hz, 1H), 8.01 (d, J = 0.8 Hz, 1H), 7.97 (d, J = 1.6 Hz, 1H), 7.82 (d, J = 8.5 Hz, 2H), 7.72 – 7.69 (m, 2H), 7.52 (s, 2H), 7.42 (dd, J = 7.9, 1.6 Hz, 1H), 7.35 (d, J = 7.9 Hz, 2H), 7.25 – 7.24 (m, 2H), 2.43 (s, 3H), 1.4 (s, 9H), 1.36 (s, 9H) ppm. ^{13}C NMR (126 MHz, $CDCl_3$) δ 194.19, 152.64, 151.08, 147.99, 146.04, 143.49, 142.85, 142.40, 139.20, 137.54, 135.46, 135.40, 135.36, 132.64, 131.48, 130.47, 127.27, 126.21, 125.93, 124.65, 124.23, 121.64, 120.69, 119.72, 119.54, 115.69, 115.18, 111.70, 111.52, 35.36, 35.22, 31.83, 31.37, 21.86 ppm. HRMS ($MALDI^{+}$ FT-ICR, dithranol): m/z = 673.17721 $[M]^{\bullet+}$, calcd. for ($C_{40}H_{35}NO_3S_3^{\bullet+}$) m/z = 673.17736.

Compound 6

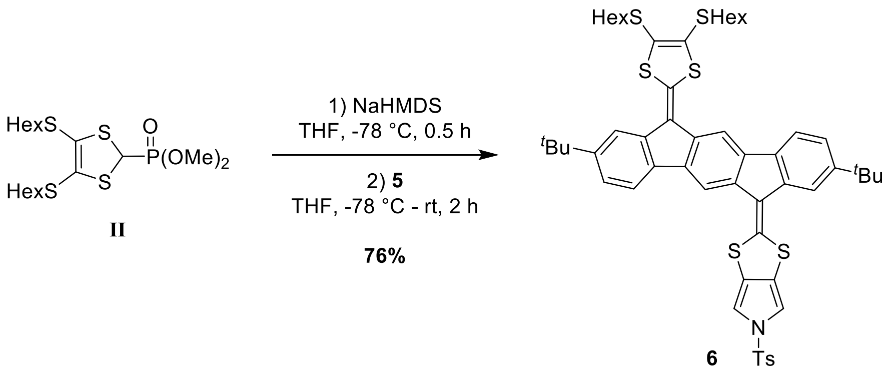

To a solution of **II** (198 mg, 0.445 mmol) in anhydrous THF (20 mL) was added NaHMDS (0.80 mL, 0.480 mmol, 0.6 M in toluene) at -78 °C, and the mixture was stirred for 0.5 h before it was added dropwise to a solution of **5** (48 mg, 0.071 mmol) in anhydrous THF (20 mL) at -78 °C. The mixture was then allowed to reach rt and stirred for a further 2 h before a sat. aq. solution of NH_4Cl (20 mL) was added, and the aqueous phase was extracted with CH_2Cl_2 (3 x 50 mL). The combined

organic phases were dried over $MgSO_4$, filtered and concentrated in vacuum. Purification by flash column chromatography (SiO_2, 20-30% EtOAc/heptane) gave **6** (54 mg, 76%) as a yellow solid. . ^{1}H NMR (500 MHz, $CDCl_3$) δ 8.30 (s, 1H), 8.05 (s, 1H), 7.98 (d, J = 1.0 Hz, 1H), 7.87 – 7.74 (m, 5H), 7.42 – 7.39 (m, 2H), 7.33 (d, J = 8.2 Hz, 2H), 7.21 – 7.16 (m, 2H), 3.13 – 2.87 (m, 4H), 2.42 (s, 3H), 1.84 – 1.65 (m, 4H), 1.46 (s, 9H), 1.46 (s, 9H), 1.37 – 1.27 (m, 12H), 0.91 – 0.88 (m, 6H) ppm. ^{13}C NMR (126 MHz, $CDCl_3$) δ 150.28, 150.20, 145.80, 142.05, 137.97, 137.82, 137.75, 137.38, 136.74, 136.23, 136.09, 135.95, 135.54, 135.51, 130.38, 129.14, 128.24, 127.20, 126.83, 126.70, 125.36, 123.81, 123.19, 121.68, 120.84, 120.16, 119.07, 119.04, 114.91, 114.15, 111.38, 111.33, 36.86, 36.79, 35.28, 35.26, 31.90, 31.88, 31.56, 31.50, 30.12, 29.99, 28.47, 22.72, 22.71, 21.84, 14.19, 14.17 ppm (one signal missing in the aliphatic region, presumably due to overlab). HRMS (MALDI$^+$ FT-ICR, dithranol): m/z = 991.27514 $[M]^{\bullet+}$, calcd. for ($C_{55}H_{61}NO_2S_7^{\bullet+}$) m/z = 991.27418.

Compound 7

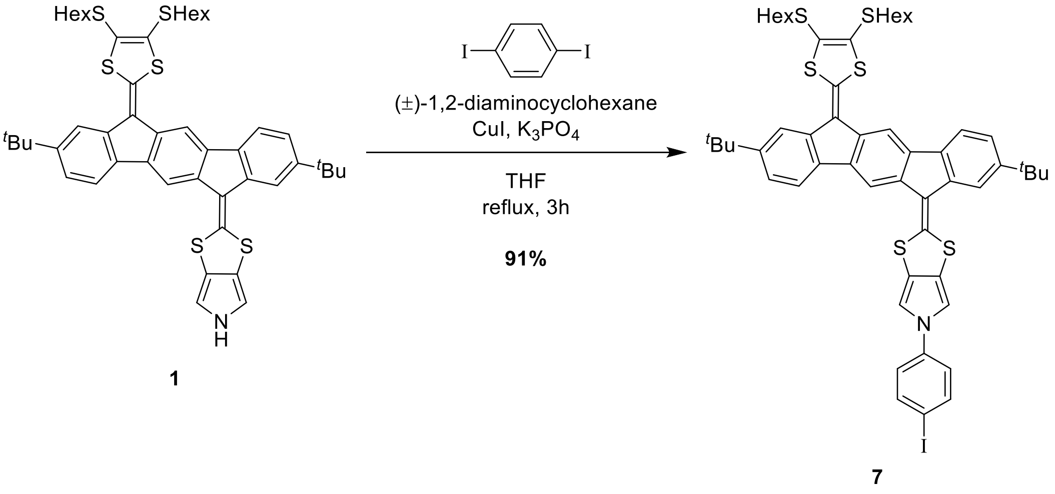

To a N_2 degassed solution of **1** (92 mg, 0.11 mmol) in anhydrous THF (10 mL) were added 1,4-diiodobenzene (13 mg, 0.039 mmol), CuI (45 mg, 0.236 mmol) and K_3PO_4 (79 mg, 0.456 mmol). The mixture was degassed for a further 30 min. before (±)-1,2-diaminocyclohexane (0.05 mL, 0.416 mmol) was added, causing a color change from yellow to dark red, then heated to reflux and stirred for 3 h before it was cooled to rt, diluted with CH_2Cl_2 (100 mL), washed with H_2O (3 x 100 mL), dried over $MgSO_4$, filtered and concentrated in vacuum. Purification by flash column chromatography (SiO_2, 40-50% CH_2Cl_2/heptane) yielded **7** (40 mg, 91% determined from 1,4-diiodobenzene) as a yellow solid. ^{1}H NMR (500 MHz, $CDCl_3$) δ 8.34 (s, 1H), 8.06 (d, J = 1.6 Hz, 1H), 8.04 (s, 1H), 7.87 (d, J = 8.0 Hz, 1H), 7.81 (d, J = 8.0 Hz, 1H), 7.77 (d, J = 1.6 Hz, 1H), 7.69 (d, J = 8.6 Hz, 2H), 7.41 (dd, J = 8.0, 1.6 Hz, 2H), 7.00 (d, J = 8.6 Hz, 2H), 6.96 - 6.93 (m, 2H), 3.03 – 2.96 (m, 4H), 1.80 – 1.73 (m, 4H), 1.50 (s, 9H), 1.48 (s, 9H), 1.36 – 1.32 (m, 12H), 0.98 – 0.89 (m, 6H) ppm. ^{13}C NMR (126 MHz, $CDCl_3$) δ 150.05, 149.99, 145.14, 139.70, 138.79, 138.18, 137.79, 137.43, 137.26, 136.36, 136.33, 135.82, 135.54, 135.38, 128.96, 128.21, 123.51, 123.13, 123.10, 122.78, 122.71, 121.83, 121.55, 120.69, 120.15, 119.07, 118.91, 114.78, 114.05, 110.17,

110.14, 90.02, 36.86, 36.78, 35.34, 35.27, 31.59, 31.53, 30.15, 30.03, 28.51, 28.50, 22.75, 22.74, 14.22, 14.19 ppm. HRMS (MALDI$^+$ FT-ICR, dithranol): *m/z* = 1039.18999 $[M]^{•+}$, calcd. for ($C_{13}H_{14}NO_5S_4^{•+}$) *m/z* = 1039.19327.

Compound 8

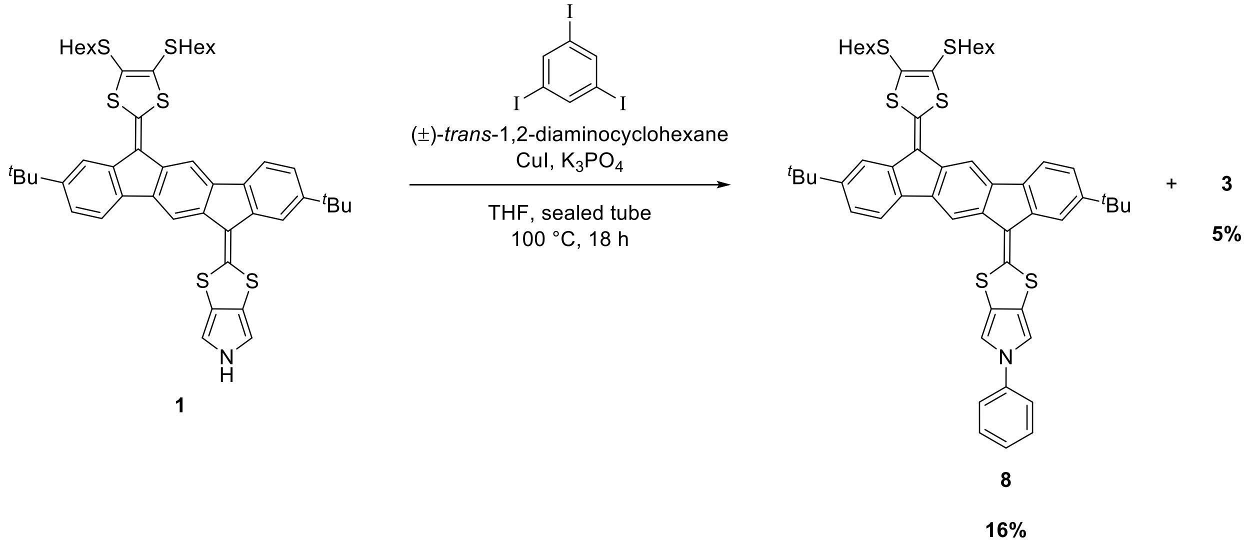

To a N_2 degassed solution of **1** (125 mg, 0.149 mmol) in anhydrous THF (7 mL) were added 1,3,5-triiodobenzene (12.5 mg, 0.027 mmol), CuI (217 mg, 1.14 mmol) and K_3PO_4 (330 mg, 1.91 mmol), and the mixture was further degassed with N_2 for 40 min. (±)-*trans*-1,2-Diaminocyclohexane (0.20 mL, 1.67 mmol) was added, and the mixture was heated to 100 °C in a sealed vial for 19 h, undergoing a color change from yellow to red, before it was cooled to rt, diluted with CH_2Cl_2 (50 mL), washed with H_2O (4 x 100 mL), dried over $MgSO_4$, filtered and concentrated in vacuum. Purification by flash column chromatography (40-100 % CH_2Cl_2/heptane) yielded **8** (22 mg, 16%) and previously isolated dimer **3** (14 mg, 5%) as yellow solids. No trace of the trimer was observed. For **8**: ^{1}H NMR (500 MHz, $CDCl_3$) δ 8.32 (s, 1H), 8.04 (d, J = 1.6 Hz, 1H), 8.01 (s, 1H), 7.84 (d, J = 8.0 Hz, 1H), 7.81 (d, J = 8.0 Hz, 1H), 7.72 (d, J = 1.6 Hz, 1H), 7.52 (t, J = 8.0 Hz, 1H), 7.43 – 7.40 (m,), 7.35 (s, 1H), 7.29 – 7.27 (m, 1H), 3.01 – 2.95 (m, 4H, 1.79 – 1.73 (m, 4H), 1.53 (s, 9H), 1.49 (s, 9H), 1.40 – 1.23 (m, 12H), 1.06 – 0.85 (m, 6H) ppm. ^{13}C NMR (126 MHz, $CDCl_3$) δ 148.41, 148.36, 143.17, 140.17, 140.17, 136.97, 136.64, 136.34, 136.09, 135.25, 135.11, 134.57, 134.50, 134.35, 130.13, 127.88, 127.26, 122.95, 122.32, 122.25, 122.23, 122.04, 120.86, 119.65, 119.07, 117.90, 117.83, 116.22, 113.78, 113.11, 110.34, 110.33, 35.86, 35.77, 33.88, 33.80, 30.80, 30.78, 30.75, 30.68, 30.62, 27.62, 21.98, 13.32 ppm (two signals missing in the aliphatic region, presumably due to overlap). HRMS (MALDI$^+$ FT-ICR, dithranol): *m/z* = 913.29978 $[M]^{•+}$, calcd. for ($C_{54}H_{59}NS_6^{•+}$) *m/z* = 913.29663.

NMR spectra of all synthesized compounds

Compound 1

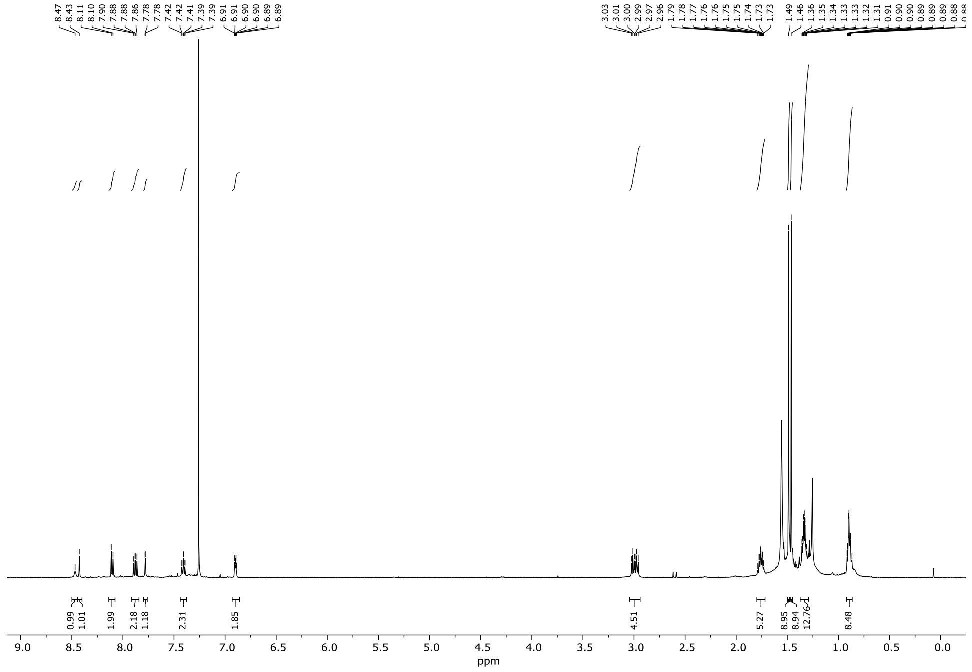

Figure S1: ^{1}H-NMR ($CDCl_3$, 500 MHz) spectrum of **1**.

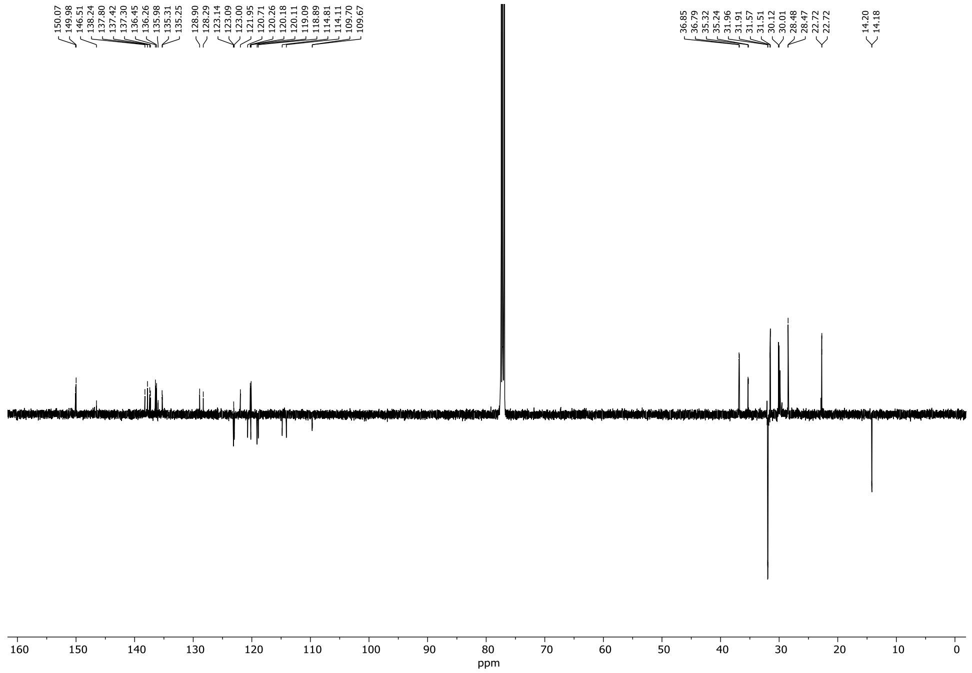

Figure S2: ^{13}C-APT NMR ($CDCl_3$, 126 MHz) spectrum of **1**.

Compound 2

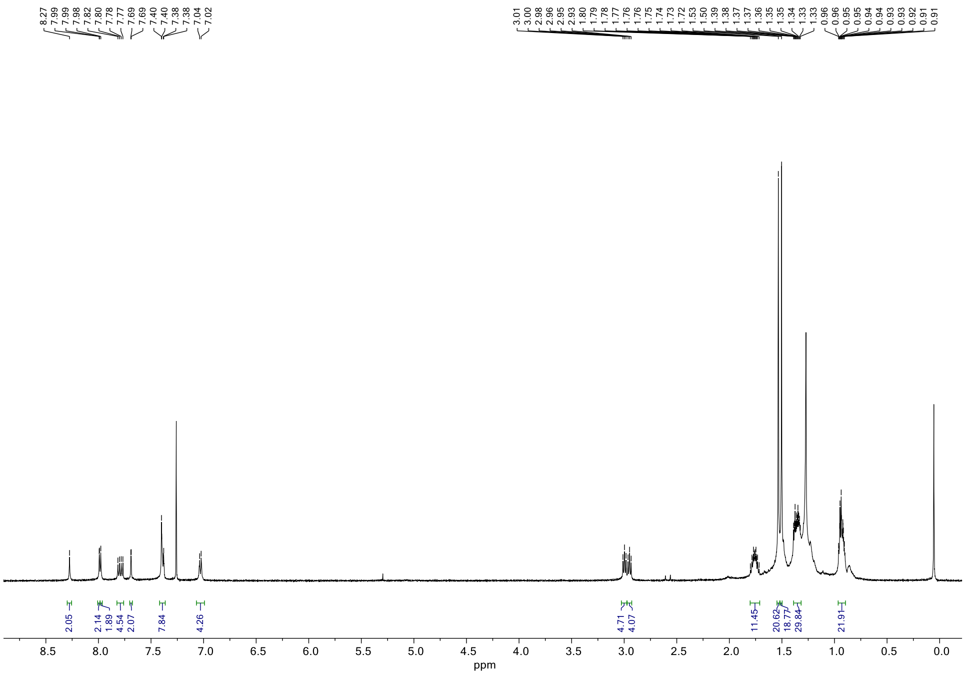

Figure S3: ^{1}H-NMR (CS_2/$CDCl_3$ (1:5), 500 MHz) spectrum of **2**.

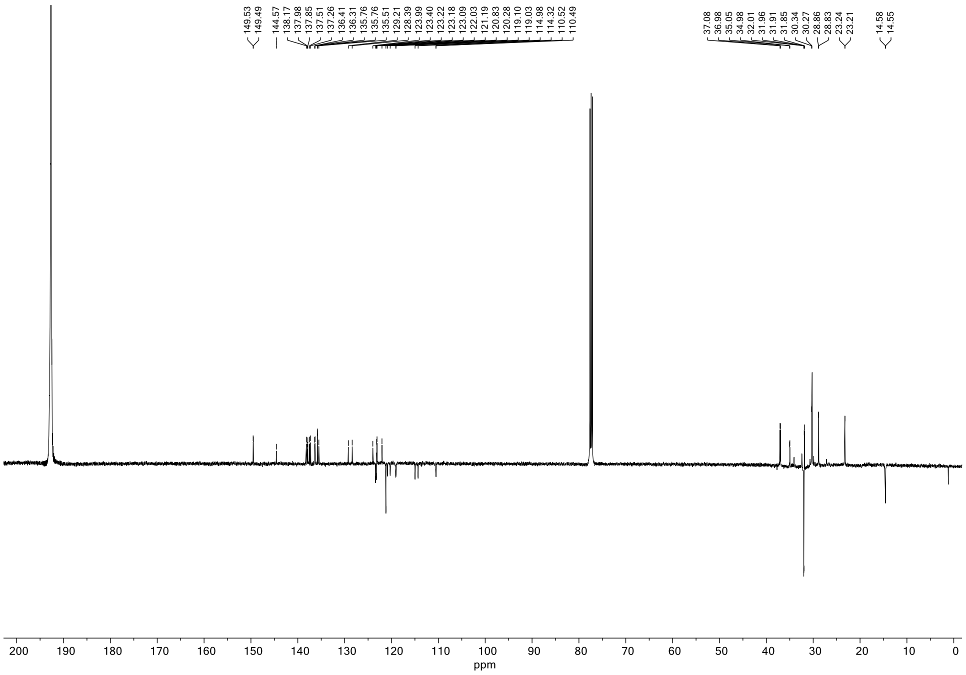

Figure S4: ^{13}C-APT NMR (CS_2/$CDCl_3$ (1:5), 126 MHz) spectrum of **2**.

Compound 3

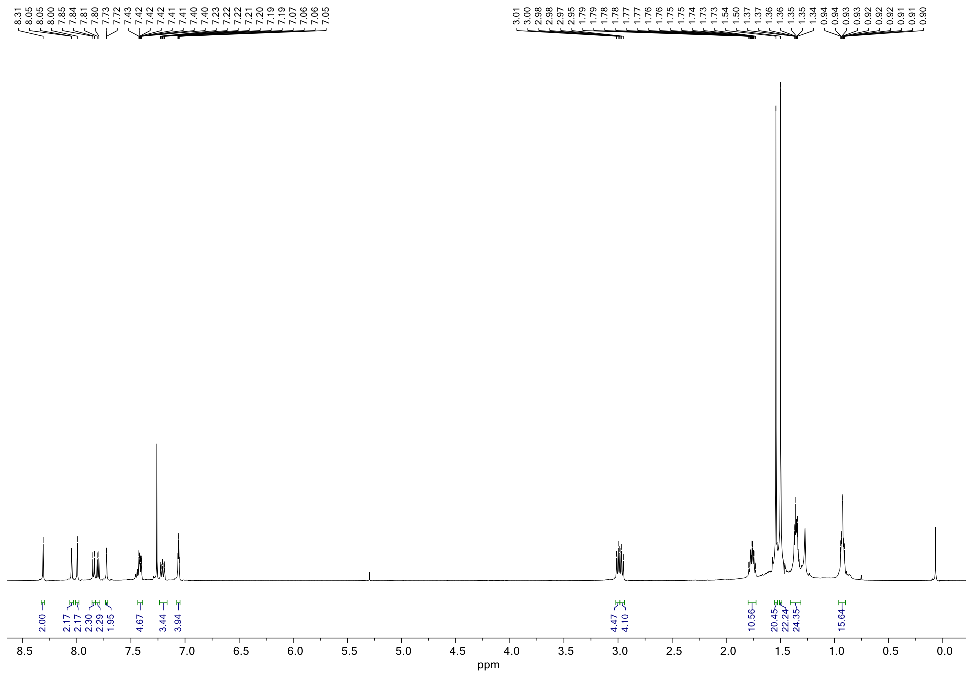

Figure S5: ^{1}H-NMR (CS_2/$CDCl_3$ (1:30), 500 MHz) spectrum of **3**.

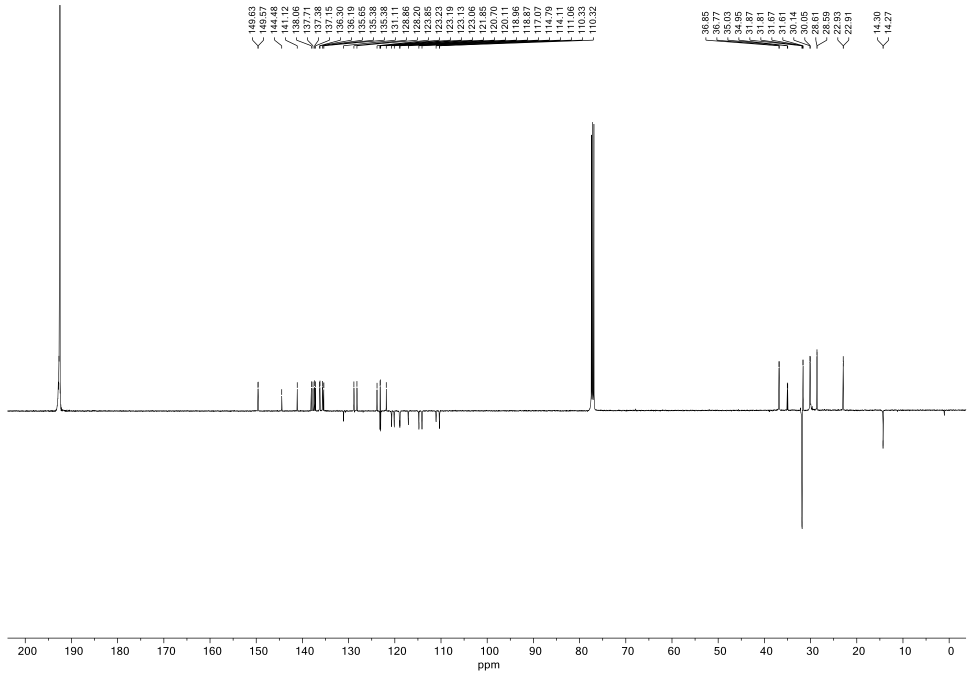

Figure S6: ^{13}C-APT NMR (CS_2/$CDCl_3$ (1:30), 126 MHz) spectrum of **3**.

Compound 5

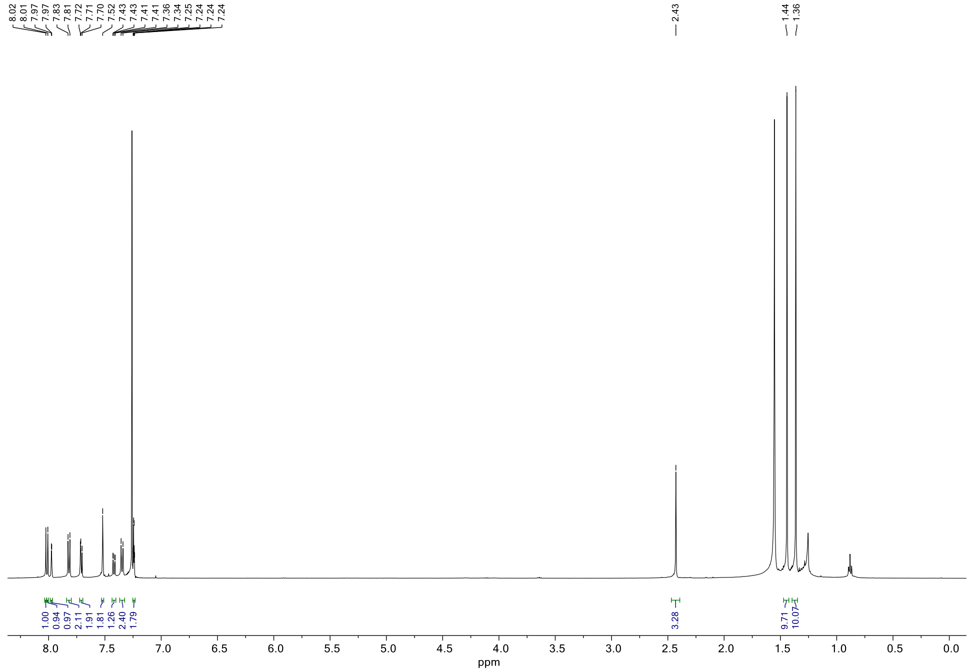

Figure S7: ^{1}H-NMR ($CDCl_3$, 500 MHz) spectrum of **5**.

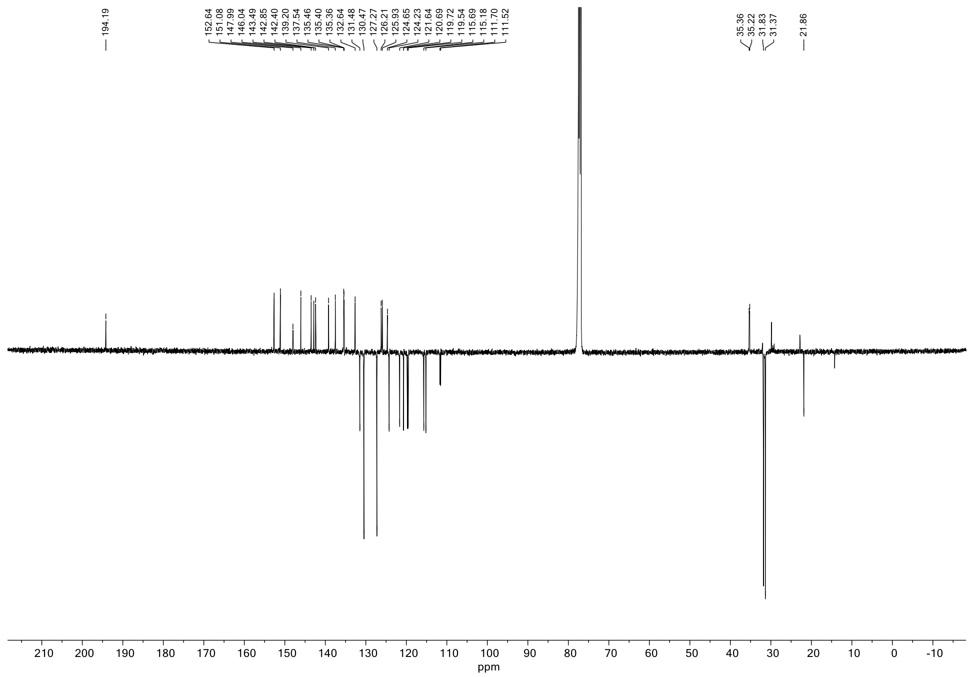

Figure S8: ^{13}C-APT NMR ($CDCl_3$, 126 MHz) spectrum of **5**.

Compound 6

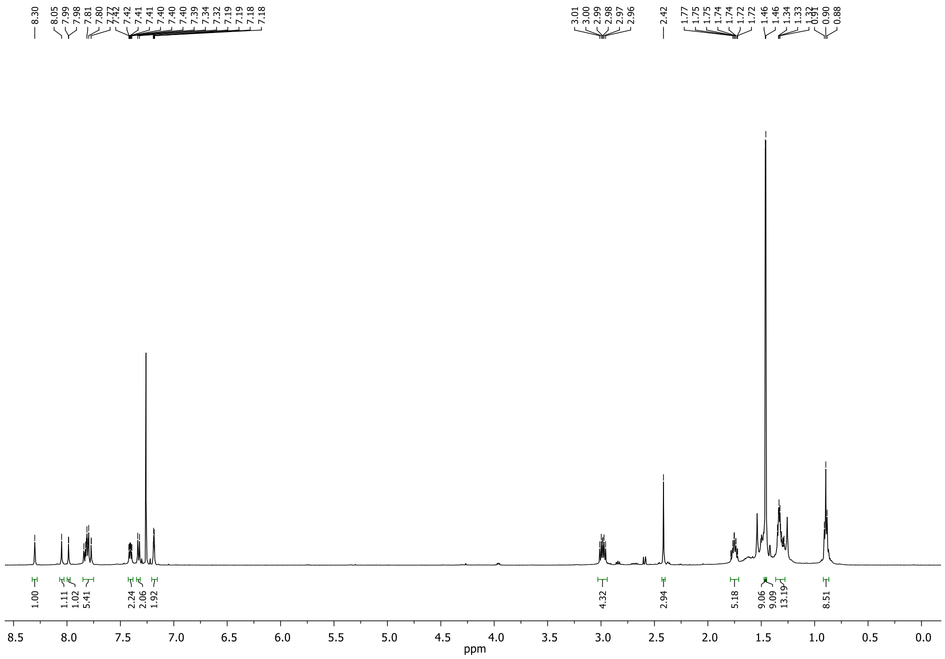

Figure S9: ^{1}H-NMR ($CDCl_3$, 500 MHz) spectrum of **6**.

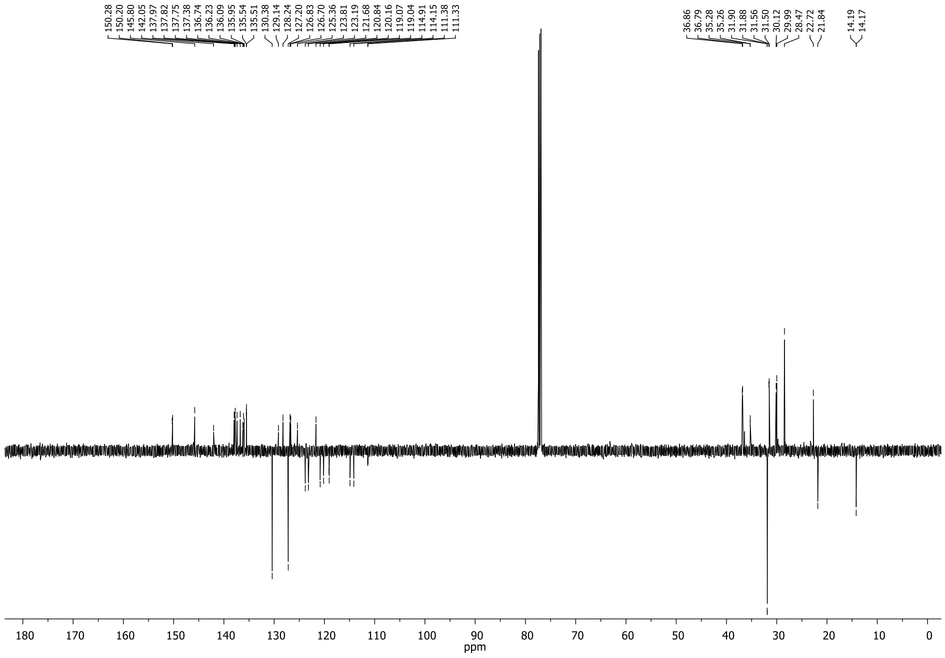

Figure S10: ^{13}C-APT NMR ($CDCl_3$, 126 MHz) spectrum of **6**.

Compound 7

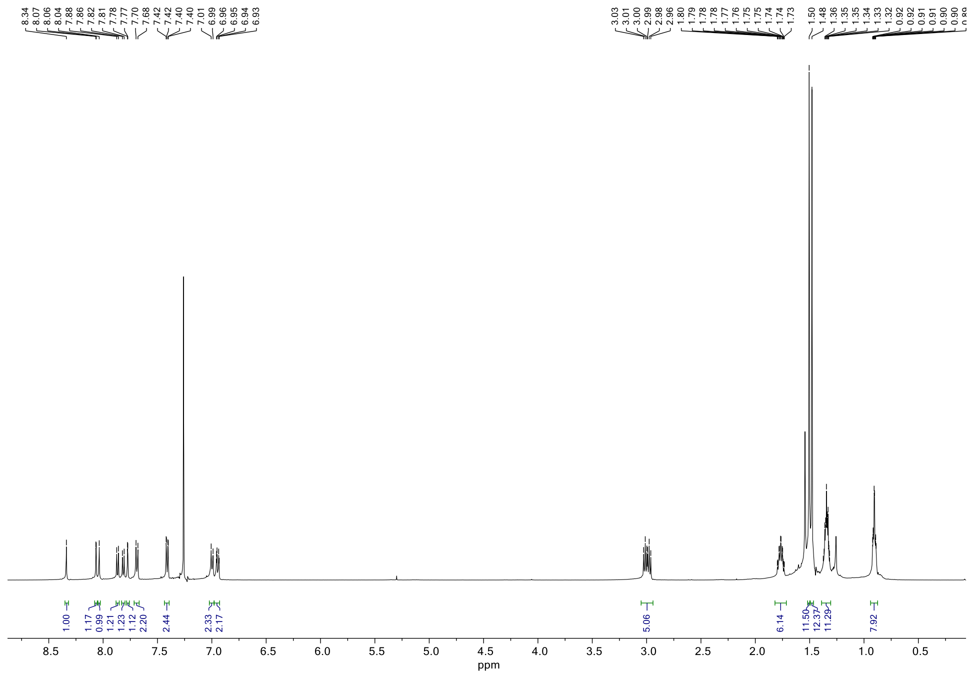

Figure S11: ^{1}H-NMR ($CDCl_3$, 500 MHz) spectrum of **7**.

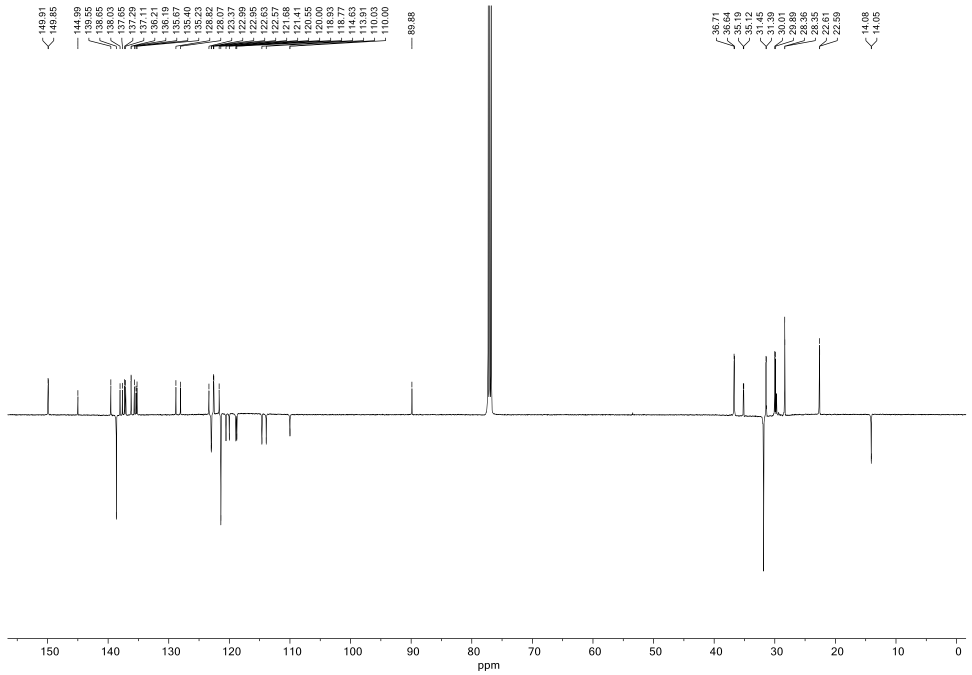

Figure S12: ^{13}C-APT NMR ($CDCl_3$, 126 MHz) spectrum of **7**.

Compound 8

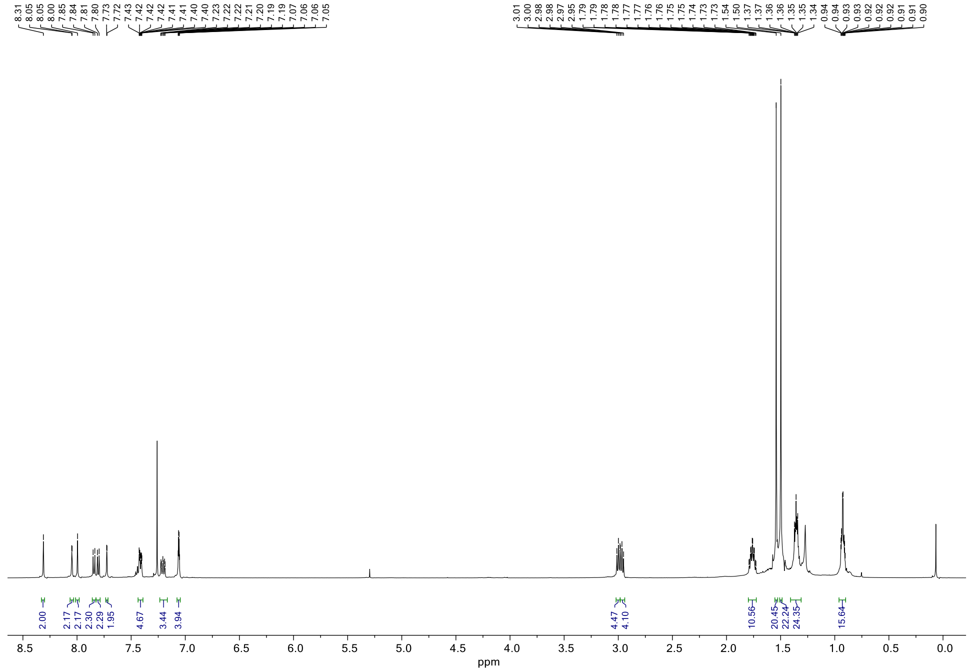

Figure S13: ^{1}H-NMR (CS_2/$CDCl_3$ (1:30), 500 MHz) spectrum of **8**.

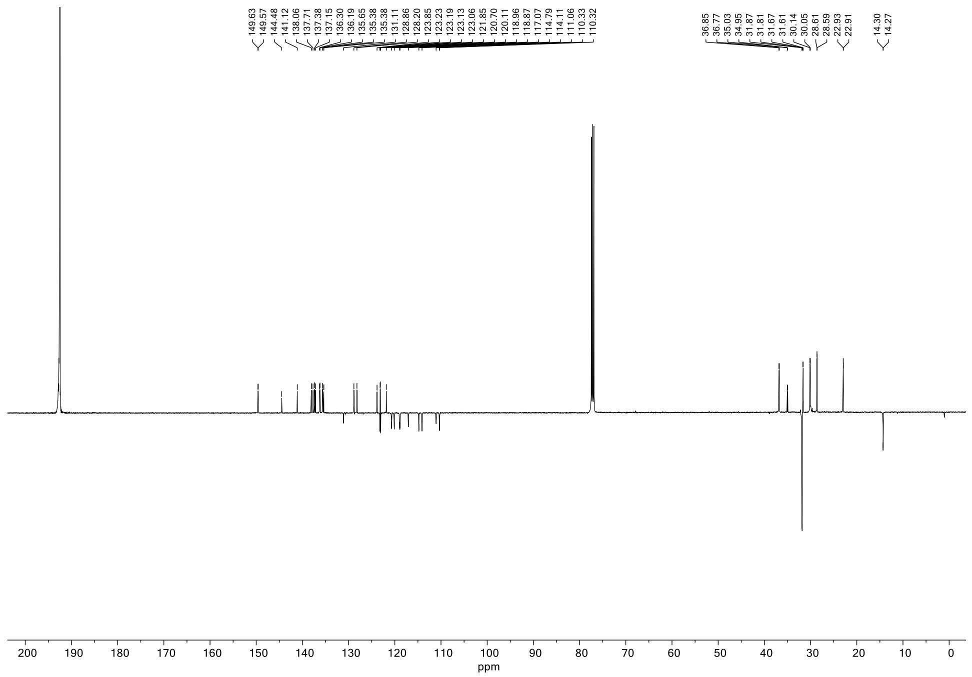

Figure S14: ^{13}C-APT NMR (CS_2/$CDCl_3$ (1:30), 126 MHz) spectrum of **8**.

Full UV-Vis spectra

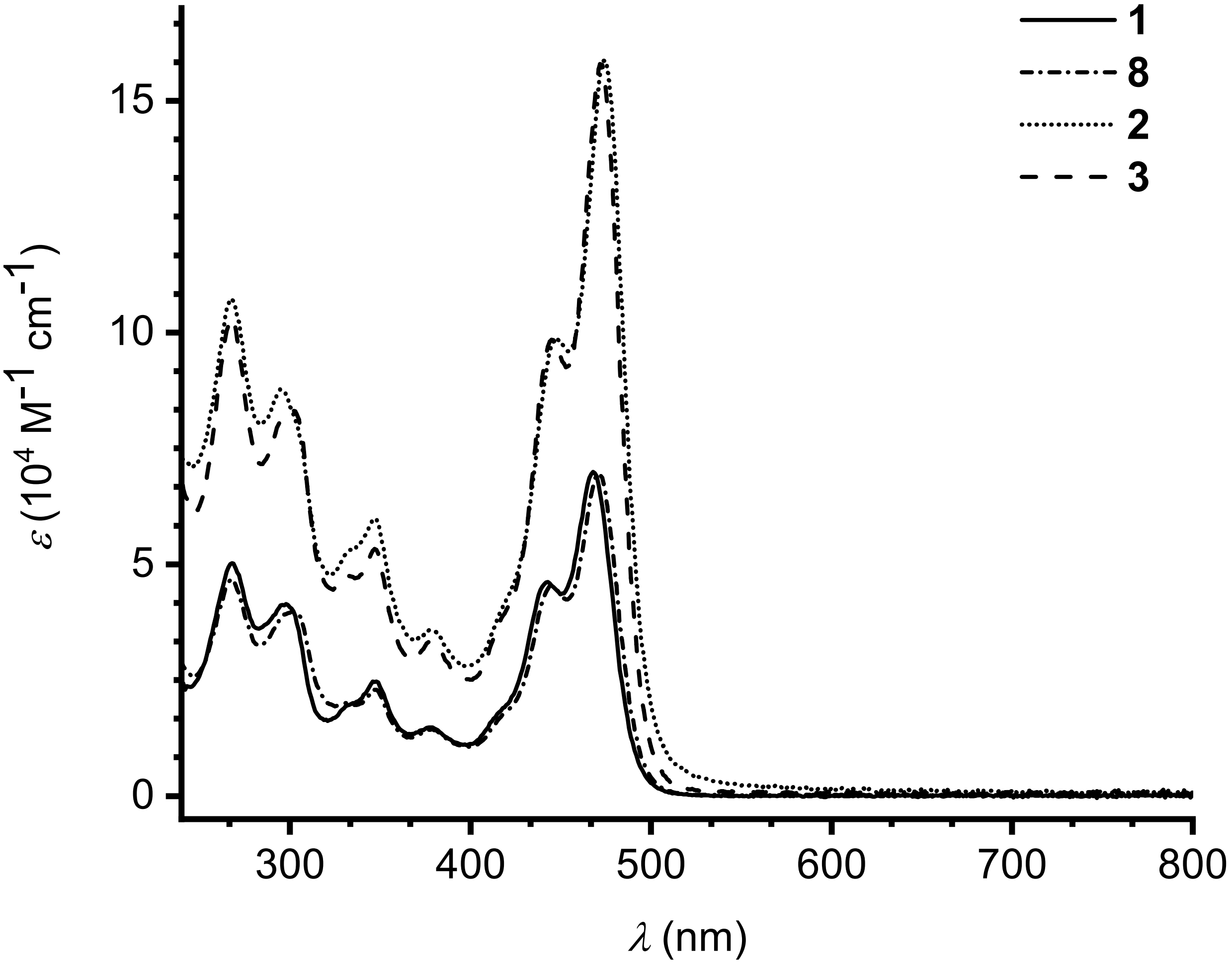

Figure S15: UV-Vis absorption spectra of compounds **1**, **8**, **2** and **3** recorded in CH_2Cl_2 at 25 °C.

Electrochemical studies

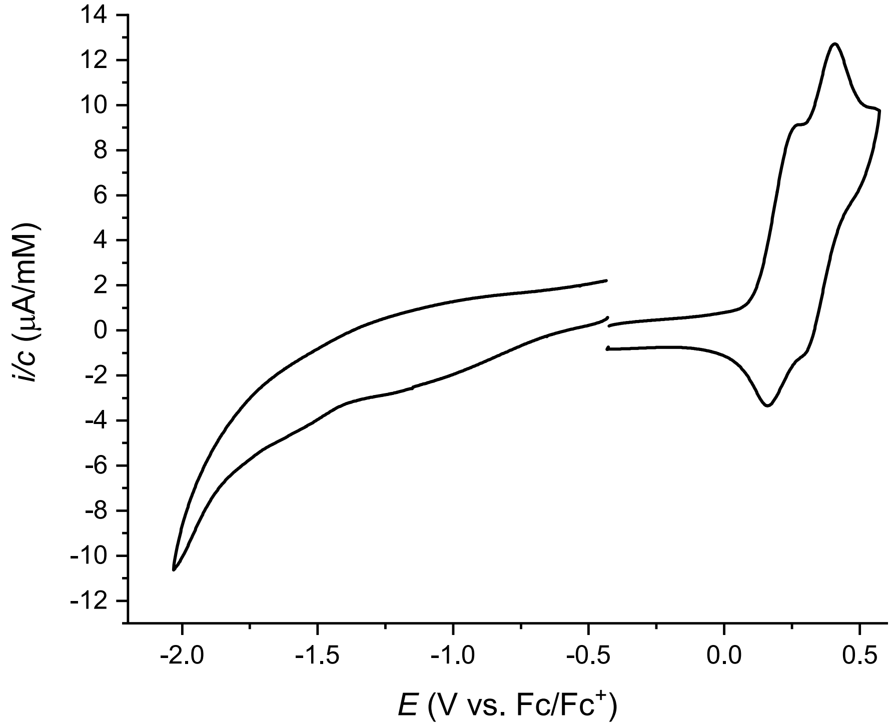

Figure S16: Cyclic voltammogram of **1** recorded in CH_2Cl_2/C_6H_5Cl (1:1). Electrolyte: 0.1 M NBu_4PF_6, scan rate: 0.1 V/s.

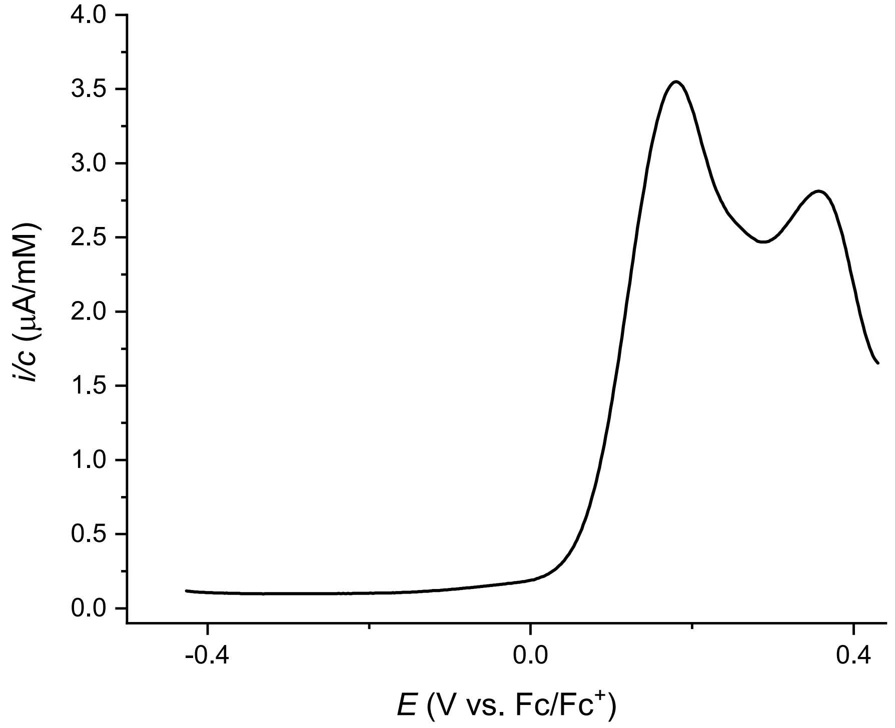

Figure S17: Differential pulse voltammogram of **1** recorded in CH_2Cl_2/C_6H_5Cl (1:1). Electrolyte: 0.1 M NBu_4PF_6, scan rate: 0.1 V/s.

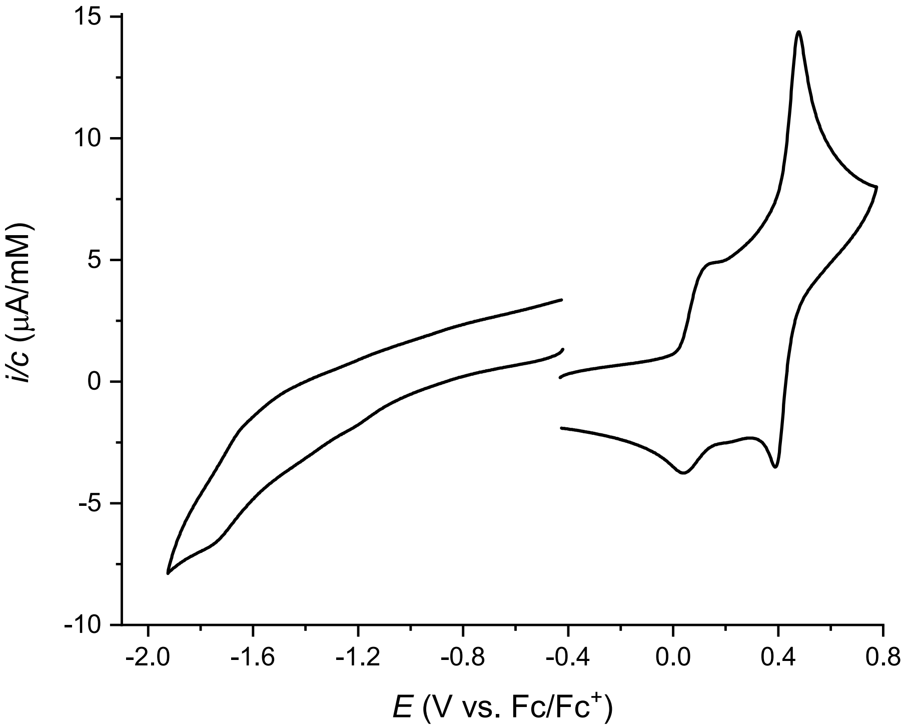

Figure S18: Cyclic voltammogram of **2** recorded in CH_2Cl_2/C_6H_5Cl (1:1). Electrolyte: 0.1 M NBu_4PF_6, scan rate: 0.1 V/s.

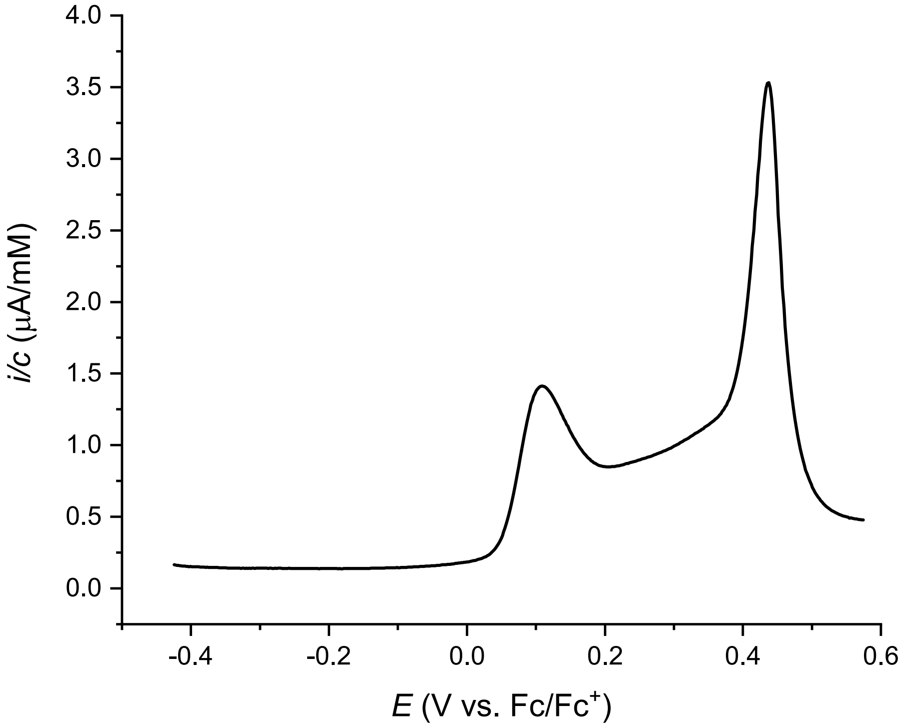

Figure S19: Differential pulse voltammogram of **2** recorded in CH_2Cl_2/C_6H_5Cl (1:1). Electrolyte: 0.1 M NBu_4PF_6, scan rate: 0.1 V/s.

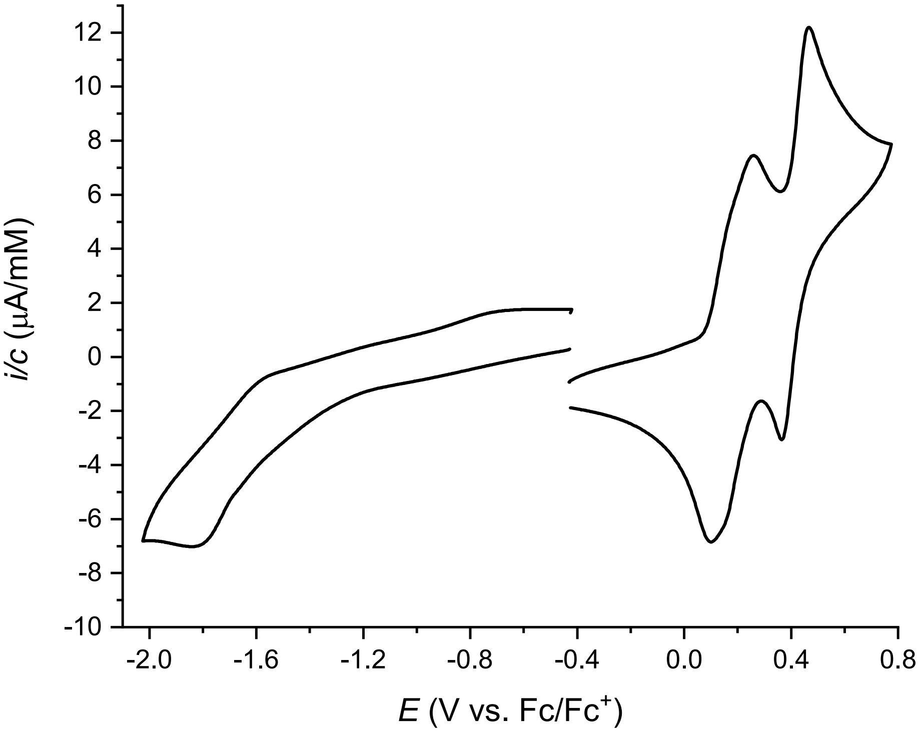

Figure S20: Cyclic voltammogram of **3** recorded in CH_2Cl_2/C_6H_5Cl (1:1). Electrolyte: 0.1 M NBu_4PF_6, scan rate: 0.1 V/s.

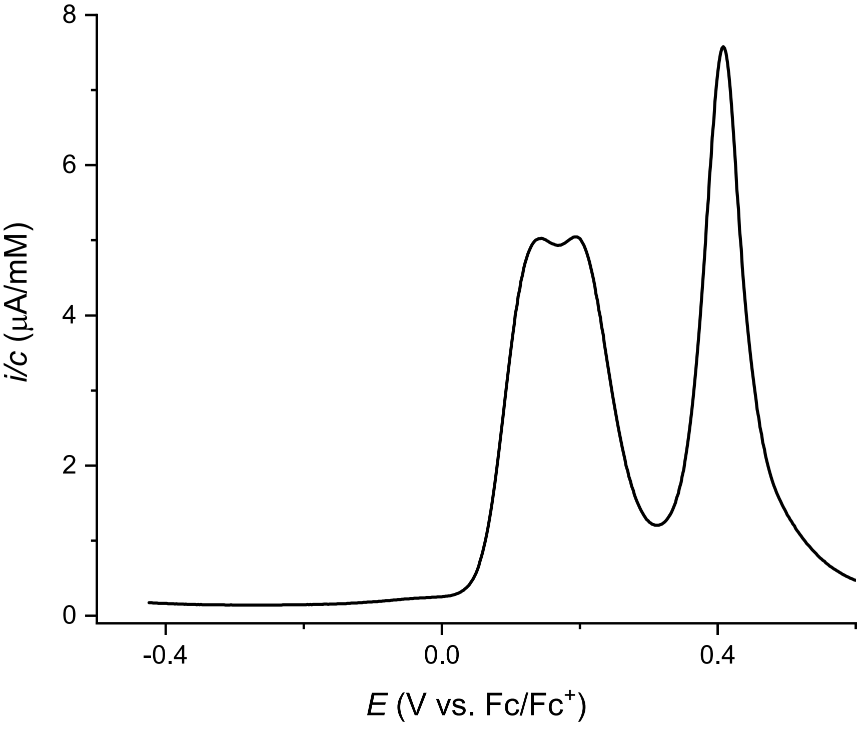

Figure S21: Differential pulse voltammogram of **3** recorded in CH_2Cl_2/C_6H_5Cl (1:1). Electrolyte: 0.1 M NBu_4PF_6, scan rate: 0.1 V/s.

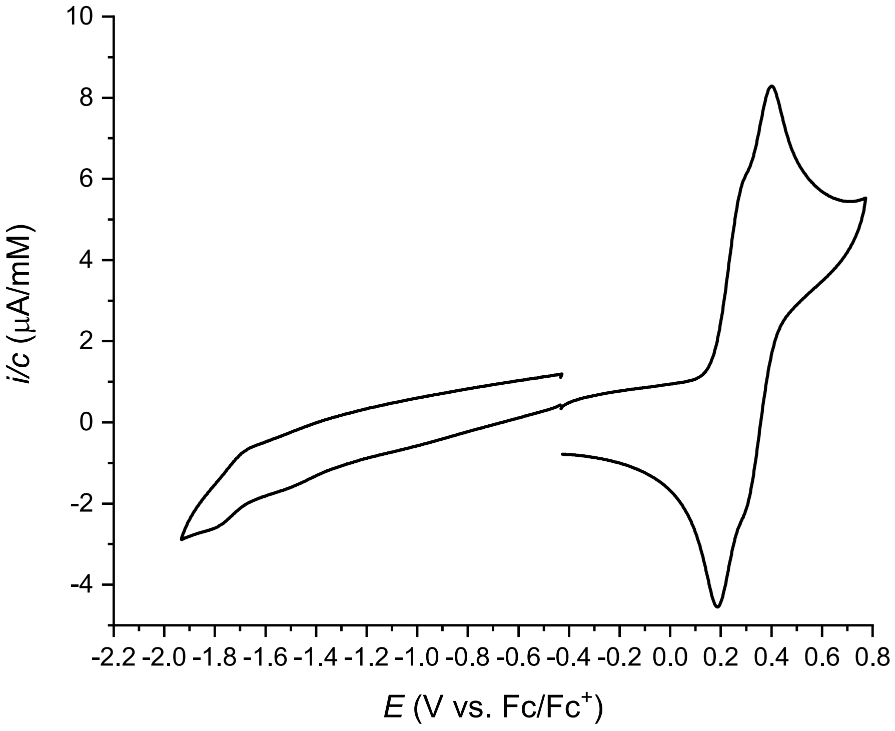

Figure S22: Cyclic voltammogram of **8** recorded in CH_2Cl_2/C_6H_5Cl (1:1). Electrolyte: 0.1 M NBu_4PF_6, scan rate: 0.1 V/s.

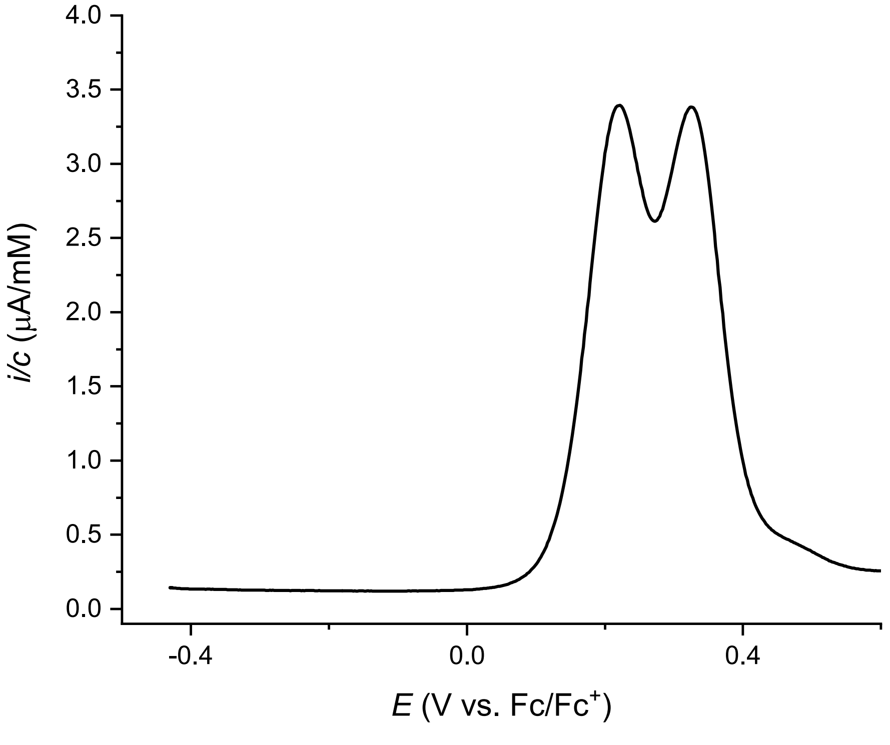

Figure S23: Differential pulse voltammogram of **8** recorded in CH_2Cl_2/C_6H_5Cl (1:1). Electrolyte: 0.1 M NBu_4PF_6, scan rate: 0.1 V/s.
